# Structure, Function, and Regulation of LytA: The *N*-Acetylmuramoyl-l-alanine Amidase Driving the “Suicidal Tendencies” of *Streptococcus pneumoniae*—A Review

**DOI:** 10.3390/microorganisms13040827

**Published:** 2025-04-05

**Authors:** Ernesto García

**Affiliations:** Centro de Investigaciones Biológicas Margarita Salas, Consejo Superior de Investigaciones Científicas, 28040 Madrid, Spain; e.garcia@cib.csic.es

**Keywords:** *Streptococcus pneumoniae*, *lytA* gene, structure and function, regulation, virulence, vaccines

## Abstract

*Streptococcus pneumoniae* (pneumococcus) is a significant human pathogen responsible for a range of diseases from mild infections to invasive pneumococcal diseases, particularly affecting children, the elderly, and immunocompromised individuals. Despite pneumococcal conjugate vaccines having reduced disease incidence, challenges persist due to serotype diversity, vaccine coverage gaps, and antibiotic resistance. This review highlights the role of LytA, a key autolysin (*N*-acetylmuramoyl-l-alanine amidase), in pneumococcal biology. LytA regulates autolysis, contributes to inflammation, and biofilm formation, and impairs bacterial clearance. It also modulates complement activation, aiding immune evasion. LytA expression is influenced by environmental signals and genetic regulation and is tied to competence for genetic transformation, which is an important virulence trait, particularly in meningitis. With the increase in antibiotic resistance, LytA has emerged as a potential therapeutic target. Current research explores its use in bacteriolytic therapies, vaccine development, and synergistic antibiotic strategies. Various compounds, including synthetic peptides, plant extracts, and small molecules, have been investigated for their ability to trigger LytA-mediated bacterial lysis. Future directions include the development of novel anti-pneumococcal interventions leveraging LytA’s properties while overcoming vaccine efficacy and resistance-related challenges. Human challenge models and animal studies continue to deepen our understanding of pneumococcal pathogenesis and potential treatment strategies.

## 1. Introduction

*Streptococcus pneumoniae* (pneumococcus) is a major human pathogen that typically colonizes the mucosal surfaces of the upper respiratory tract asymptomatically (carrier state). Carriage is a prerequisite for the development of pneumococcal disease [1,2]. Although traditionally regarded as a strictly extracellular bacterium, increasing evidence suggests that pneumococcus can also exist in an intracellular state [3,4,5,6,7,8]. Pneumococcus is a common cause of non-invasive conditions such as otitis, conjunctivitis, and pneumonia, as well as life-threatening invasive pneumococcal diseases (IPD), including sepsis, bacteremic pneumonia, and meningitis, particularly in children, the elderly, and immunocompromised individuals. Along with *Neisseria meningitidis* and *Haemophilus influenzae* type b, *S. pneumoniae* is responsible for over 70% of meningitis cases (>100,000 episodes) documented over an 80-year period, and its prevalence has increased in recent years [9]. Globally, the colonization rate of *S. pneumoniae* is estimated to average 1.9–5.8 billion individuals at any given time [10]. Additionally, in 2021, pneumococci accounted for more than 90 million pneumonia cases and 450,000 deaths worldwide [11].

Pneumococcal conjugate vaccines (PCV) have significantly reduced the burden of IPD [12]. However, the impact of PCV on pneumococcal carriage remains uncertain, with conflicting findings being reported [13]. The high diversity of pneumococcal serotypes (over 100 identified), limited serotype coverage, and serotype replacement by non-PCV13 strains present ongoing challenges [14]. Furthermore, regional disparities in vaccine coverage persist, ranging from 86% in the European region to only 26% in the Western Pacific region. Globally, the World Health Organization (WHO) estimates that 40% of children under 5 years old remain unprotected by PCV [15]. The severity of pneumococcal disease is largely attributed to a robust inflammatory response triggered by complement activation and cytokine release [16]. These responses are elicited by bacterial components such as capsular polysaccharides, surface proteins, or DNA released as bacterial byproducts.

Antibiotic resistance poses a significant global health threat and is projected to cause 10 million deaths annually by 2050 if current trends of inappropriate and excessive antibiotic use continue [17]. Of particular concern is the emergence of multidrug-resistant (MDR) *S. pneumoniae* strains, which are resistant to β-lactams, macrolides, fluoroquinolones, and sulfamethoxazole/trimethoprim [18]. In the 2024 Bacterial Priority Pathogens List, the WHO categorized *S. pneumoniae* as a medium-priority pathogen, emphasizing the urgent need to address its public health impact, particularly in vulnerable populations within resource-limited settings [19].

The extensive research on *S. pneumoniae* makes it challenging to cover all aspects of this pathogen comprehensively. Since 2011, approximately 14,000 articles mentioning “*Streptococcus pneumoniae*” have been added to the PubMed database (https://pubmed.ncbi.nlm.nih.gov/?term=Streptococcus+pneumoniae&sort=date (accessed on 25 March 2025)), reflecting the broad interest in studying various aspects of the biology of this microorganism. This review aims to summarize current knowledge, focusing on the major pneumococcal autolysin and addressing new, open questions such as the role of pneumococcal prophages in autolysin evolution. However, many other important topics and references in related fields will not be covered. For additional details, readers are encouraged to consult comprehensive reviews on the history of pneumococcal research, pathogenesis, virulence factors and host immunity, genomics and genetics, or vaccine development [5,20,21,22,23,24,25,26,27,28,29,30,31,32,33,34,35,36,37].

- *Streptococcus pneumoniae* is a major pathogen that colonizes the upper respiratory tract and can cause serious invasive diseases. While vaccines have reduced disease burden, issues like serotype diversity, limited coverage, and antibiotic resistance remain. The WHO classifies it as a medium-priority pathogen. This review examines key biological aspects of the pneumococcal autolysin LytA, including its regulation and control, role in virulence, therapeutic potential, and evolutionary implications.

## 2. The “Suicidal Tendencies” of *S. pneumoniae*

In 1890, shortly after the isolation of *S. pneumoniae* by Pasteur et al. [38] and Sternberg [39], Welch noted that the resolution of pneumococcal exudate in empyema fluid following pneumonia was accompanied by the lysis of pneumococci within their capsules, a process observable microscopically [40]. In 1900, Neufeld [41] first reported the rapid lysis of pneumococci induced by bile or bile salts, which was attributed to their detergent action (for a thorough overview of early pneumococcal studies, refer to [42]). Sodium deoxycholate (Doc) is currently used as a replacement for bile [43,44,45,46]. Notably, Doc also kills pneumococci—but not other streptococci—by a, still unknown, non-autolytic mechanism [47]. The first detailed description of the autolytic process was likely provided by Rosenow in 1910 [48], who demonstrated that pneumococci disintegrate when suspended in physiological saline. This autolysis was neither due to the action of NaCl nor the solubility of pneumococci in water. Subsequent studies revealed that pneumococci possess bacteriolytic intracellular enzymes capable of lysing heat-killed pneumococci, with optimal activity at pH 6–8 [49,50].

**Figure 1:**
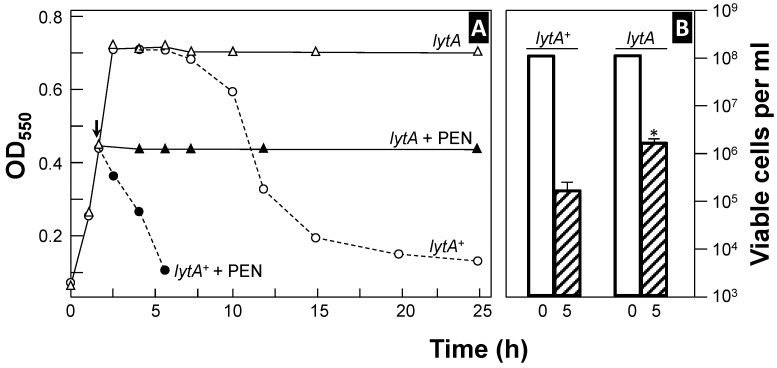
Curves of growth and penicillin-induced lysis of two pneumococcal strains (panel (**A**)). Pneumococci were incubated in a semisynthetic medium at 37 °C, and their growth (and lysis) was monitored by measuring optical density at 550 nm (OD_550_). At the time indicated by the arrow, the samples from the same cultures were treated with penicillin (PEN, 100 × MIC), and incubation was continued at the same temperature (solid symbols). Panel (**B**) shows the viability of the cultures at the time of antibiotic addition (open bars) and after 5 h of incubation (hatched bars). The asterisk indicates statistically a significant difference (*p* < 0.001) compared to the results for the wild-type, *lytA*^+^ strain.

Autolysis during the stationary phase is more pronounced when pneumococci are incubated at 37 °C in a semi-synthetic or chemically defined medium (CDM) compared to rich media [51]. However, this phenomenon varies between strains; for example, strain TIGR4 readily undergoes autolysis in a rich medium but often does not in a CDM [52]. This distinctive autolytic behavior has been termed the “suicidal tendency” of pneumococci [53]. As expected, autolysis is concomitant with viability loss (Figure 1). However, spontaneous death at the stationary phase is not only due to autolysis but also the production of hydrogen peroxide (H_2_O_2_) [54]. Moreover, it should be underlined that false-positive blood cultures may result from the autolysis of *S. pneumoniae* in the culture medium [55,56]. Notably, antibiotics targeting cell wall synthesis are less lethal to pneumococcal strains deficient in autolytic activity [51,57,58,59,60]. Additionally, sitafloxacin, a fluoroquinolone with a high affinity for DNA gyrase and topoisomerase enzymes, exhibits strong bactericidal activity against *S. pneumoniae* by triggering the activity of LytA, the main pneumococcal autolysin [61]. Recent findings indicate that sitafloxacin treatment significantly increases the transcription and translation of the *lytA* gene encoding LytA [62] (for a complete list of the pneumococcal genes mentioned in this review, see Table S1). In addition, when grown under anaerobic conditions, pneumococcal autolysis is inhibited [63], and in contrast with microaerophilic conditions, the transcription of *lytA* under anaerobiosis was not altered upon entry into the stationary phase of growth [64].

LytA, an *N*-acetylmuramoyl-l-alanine amidase (NAM-amidase; EC 3.5.1.28) [65,66], is the primary autolytic enzyme in *S. pneumoniae* and is responsible for both autolysis at the end of the exponential phase and Doc- or penicillin (PEN)-induced lysis [59,60,67,68,69]. LytA activity is optimal at 37 °C. To date, the *lytA* gene is universally employed for accurate qPCR-based identification of pneumococcal carriage [44,70].

Another autolytic enzyme, LytC lysozyme (EC 3.2.1.17), has also been identified, acting primarily at 30 °C [71,72]. Similarly to LytA, LytC contributes to the bactericidal effect of PEN, but only under conditions mimicking the temperature of the upper respiratory tract (approximately 30–34 °C) [71,73,74,75].

A unique case of pneumococcal autolysin is CbpD, a peptidoglycan (PG) hydrolase defined as an enzyme that induces self-lysis [76]. Unlike LytA and LytC, CbpD is secreted and plays a role in “fratricide” or “allolysis”. Allolysis is a killing mechanism that could be used by competent cells to acquire DNA from non-competent pneumococci; CbpD lyses non-competent sister cells in collaboration with LytA and LytC in liquid cultures [77,78,79,80]. Competence for genetic transformation is a physiological state that enables the uptake of exogenous DNA. Moreover, induction of competence for genetic transformation is a general response to stress in Gram-positive bacteria (for reviews see [22,35]). Allolysis also involves a previously undescribed bacteriocin system consisting of a two-peptide bacteriocin, CibAB, and its immunity factor, CibC [78]. CibAB alone cannot induce cell lysis but may function as a trigger factor for fratricide. Competent attacker cells are protected from CbpD-mediated lysis through the production of the immunity protein ComM, which also promotes a transient division delay [81,82]. While CbpD alone is insufficient to induce substantial lysis in mixed cultures, the addition of a chelating agent (e.g., EDTA) enhances fratricidal efficiency [83]. Although not confirmed yet, CbpD likely cleaves amide or peptide bonds in pneumococcal PG stem peptides in conjunction with LytA and LytC [80,84]. On the other hand, competent biofilm cells of *S. pneumoniae* undergo transformation more efficiently from neighboring cells than from DNA present in the growth medium. Effective lysis of target cells necessitates the cooperative action of CbpD and LytC, while LytA is not required for efficient gene exchange in the biofilm environment [85]. Of note, among the five genes involved in fratricide (*lytA*, *lytC*, *cbpD*, *cibA*, and *cibB*), genetic epistasis analyses indicated that LytA is the most dominant allolytic enzyme during pneumococcal pathogenesis in a mouse model of infection [86].

LytA, in addition to driving the suicidal tendencies of *S. pneumoniae*, has been attributed to three key roles in pneumococcal biology. First, it catalyzes the separation of the daughter cells at the end of the cell division to produce “diplo” cells [59,60]. Second, as a key virulence factor, LytA releases cell wall fragments and cytoplasmic proteins during infection, which serve as inflammatory mediators [87,88,89,90,91,92,93,94,95,96,97,98,99]. Notably, tripeptides were over 100-fold more potent than intact peptidoglycan. However, it is important to note that, on a weight-to-weight basis, the whole peptidoglycan from Gram-positive bacteria is approximately 1000-fold less active than the lipopolysaccharide of Gram-negative organisms [95]. Autolysis also contributes to the release of extracellular DNA (eDNA), which is considered important for in vitro biofilm formation [100,101,102]. Confocal micrographs also showed that the biofilms formed by a *lytA* mutant were consistently thinner (15–20 μm) than those formed by the parental *lytA*^+^_strain (≥30 μm). LytA plays a variable (but additive) role in biofilm formation on abiotic surfaces. Moreover, LytA, along with other choline-binding proteins (CBPs; see Section 4), has been shown to bind eDNA [103]. This DNA binding capacity of CBPs appears to be independent of their enzymatic activity and, at least in the case of LytA, does not require the choline-binding domain (CBD) characteristic of CBPs (see Section 4.3 below). These results have been independently confirmed using different experimental approaches [104,105].

Autolysis contributes not only to reducing in vitro growth but also to pneumococcal pathogenesis by shielding bacteria from the immune system and enhancing toxin release (see Section 6.2) [106,107]. These components may interfere with opsonization or induce localized inflammation that diverts immune surveillance. Moreover, the release of eDNA and other factors may contribute to a protective matrix, thereby hindering phagocytosis. In contrast, LytA-deficient mutants do not undergo autolysis and fail to release these modulatory factors, making them more susceptible to recognition and clearance by phagocytic cells. Furthermore, when LytA was inactivated, pneumococci stimulated significantly higher production of tumor necrosis factor, and pro-inflammatory cytokines like interferon-γ or IL-12 in human peripheral blood mononuclear cells, while levels of anti-inflammatory cytokines IL-6, IL-8, and IL-10 remained unchanged. Third, in cooperation with phage-encoded lytic enzymes, LytA facilitates phage progeny release, possibly by collapsing the proton motive force across the bacterial membrane [108,109,110,111,112].

- *Streptococcus pneumoniae* autolysis, first observed in the late 19th century, is primarily driven by LytA, a key enzyme responsible for cell lysis, virulence, biofilm formation, and immune evasion. LytA also facilitates the release of inflammatory molecules and extracellular DNA, aiding pathogenesis and genetic exchange. Additional autolysins, LytC and CbpD, contribute to antibiotic response and fratricide—a process enabling DNA uptake from sibling cells. LytA is essential for complete cell separation. Its inactivation increases immune detection and reduces inflammation.

## 3. Organization of the *lytA* Gene

**Figure 2:**
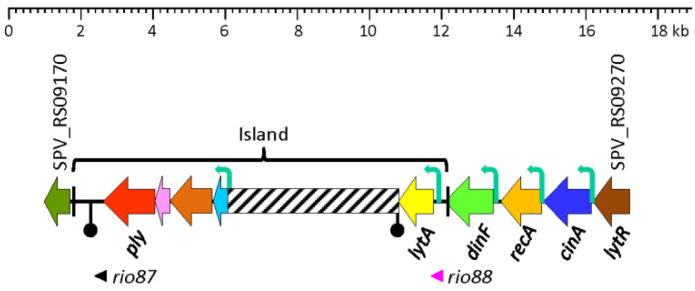
Diagram showing the region of the *S. pneumoniae* D39V genome (NZ_CP027540.1) between SPV_RS09170 and SPV_RS09270, and containing the *lytA* gene. The pathogenicity island (*ply–lytA*) is flanked by a ~100 nt direct repeat (*pl*REP; black bars). Genes are shown with arrows pointing in the direction of the transcription. The “inverted matchsticks” represent transcriptional terminators. Bent arrows show the location of functional promoters. A region which is highly variable among different *S. pneumoniae* strains is represented by a hatched bar. The sORF *rio87* (SPV_2545) has been described elsewhere [141].

The *lytA* gene is located immediately downstream of the *cinA–recA–dinF* gene cluster in the *S. pneumoniae* genome and forms part of a pathogenicity island (*ply–lytA*) which is flanked by a ~100 nt direct repeat (*pl*REP), one copy located downstream of *ply* and the other overlapping the termination codon of *dinF* [113] (Figure 2). The *ply* gene codes for the pore-forming cytotoxin, pneumolysin (Ply), a well-known *S. pneumoniae* virulence factor [114,115]. The genes *cinA*, *recA*, *dinF*, and *lytA* form an operon and encode, respectively, a competence/damage-inducible protein A [116] that may have a role in facilitating the localization of RecA to the membrane [117], the RecA recombinase [118], a member of the multidrug and toxic compound extrusion (MATE) transporter family associated with quinolone susceptibility [119] that has been reported as essential for lung infection [120], and the LytA autolysin. Different studies have shown that the *lytA* gene is transcribed from four different promoters: (1) its own constitutive promoter [121], (2) the promoter associated with *dinF*, (3) the promoter corresponding to the *recA* gene, and (4) the competence-specific promoter located upstream of *cinA*. The latter promoter contains a conserved sequence, TACGAATA, designated as the Cin box (or Com box). The alternative sigma factor ComX (σ^X^) allows the core RNA polymerase to recognize competence-specific promoters [122,123,124,125,126], which lack obvious σ^A^ (*rpoD* or s*igA*) promoter sites [127]. As ComW participates in the activation and stabilization of ComX and is required for full activity of σ^X^ in directing transcription of late competence genes, it can be assumed that ComW participates—in conjunction with ComX—in the stimulation of the transcription of *lytA*, as it happens in *comW* mutants where reduced expression of several late competence genes has been noted [128]. More recent results suggest that ComW functions as a novel σ factor activator during transformation in *S. pneumoniae* [129,130].

With respect to the constitutive promoter of *lytA*, Díaz and García [121] proposed the sequence TTGACt–17 nt–TAaAgT (consensus sequences appear in capital letters) located at a reasonable distance from the transcription start site (TSS). LytA levels remain constant before and at the onset of growth phase-dependent autolysis [121,131,132,133]. Alternatively, and as occurs for the *recA* promoter, where no obvious consensus −35 promoter signal was reported in the original publication [119], putative pneumococcal extended −10 sequences—sufficient for promoter activity in several documented cases [134]—can be found, i.e., TaTGaTATAAT for *lytA* and TtTGaTATAAT for *recA*. Recent data indicate that an additional −10 extended promoter (gcTGaTATAAT) may be located at 5′ of the initiation codon of *dinF* and that the corresponding transcript also includes *lytA* [135,136].

Notwithstanding an imperfect terminator located at 3′ of *recA* [135], only one high-confidence transcriptional terminator is located at 3′ of *lytA* on the whole *cinA–recA–dinF–lytA* operon [121]. The TSS of *lytA* was found 240 nt upstream of the initiation codon (ATG) of the gene (position 1,730,799 in the D39 genome). This long leader sequence, which potentially may encode two small proteins, was conjectured to be somehow involved in regulating the synthesis of the NAM-amidase [121] but, unfortunately, no experimental evidence for that assumption was available at that time. A recent study has reported that a small RNA (sRNA), SPD_sr95, is transcribed between positions 1,730,706 and 1,730,807 from the minus strand of the D39 chromosome [137]. Remarkably, polyribonucleotide nucleotidyltransferase (EC 2.7.7.8) (also named polynucleotide phosphorylase or PNPase) influences the levels of a large number of regulatory sRNAs. In particular, the transcript level of SPD_sr95 decreased by ~2.5-fold in a Δ*pnp* mutant compared to the *pnp*^+^ parent strain during exponential growth in rich (BHI) broth [138]. Evidence indicating any defect on autolysis was not obtained since the Δ*pnp* mutant did not show any detectable defect in vitro (although it was attenuated in vivo). To date, only a few small proteins have been characterized but, in those cases, they have shown many important and varied functions (for reviews, see [139,140]).

**Figure 3:**
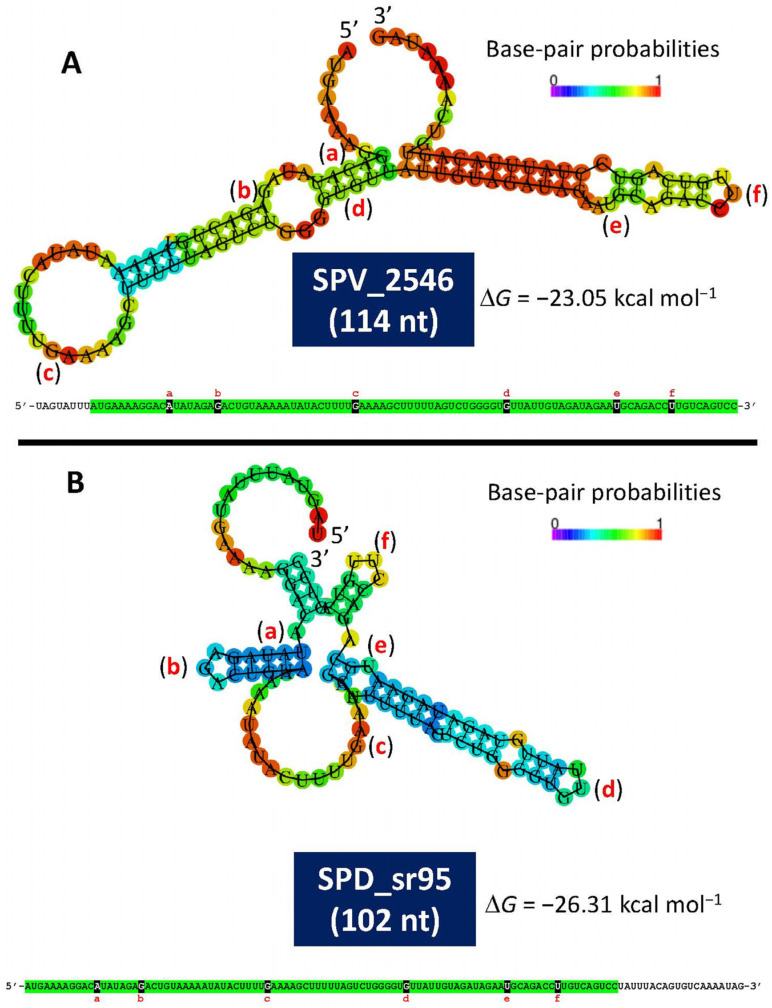
Minimum free energy and secondary structure of the complementary sequence of SPV_2546 (**A**) and SPD_sr95 (**B**), as predicted by the RNAfold WebServer (http://rna.tbi.univie.ac.at/cgi-bin/RNAWebSuite/RNAfold.cgi (accessed on 25 March 2025)). The corresponding nucleotide sequences are also shown. Identical sequences are highlighted in green. Letters a to f indicate identical nucleotide positions in both RNA sequences.

A recent study has provided experimental evidence of the existence of a small open reading frame (sORF) (locus tag SPV_2546; strain D39V) located in the *lytA* promoter-containing region [141]. This ORF encodes a 37-residues-long polypeptide, identical to one of the two small proteins predicted in 1990 [121]. This ORF, designated as *rio88* (for Ribo-seq-identified ORFs), is likely expressed in every pneumococcal isolate. The location of SPV_2546/*rio88* in the equivalent D39 genome is: 1,730,666–1,730,799 and is also encoded in the minus strand. It must be noted that SPV_2546 (114 bp) and SPD_sr95 (102 nt) do not completely overlap. Whether this is correct or simply represents an experimental artifact is not known yet. Notably, small proteins nearly identical to SPV_2546 are predicted to be encoded by any other *S. pneumoniae* genome analyzed (unpublished observations). In addition, a prediction of the secondary structures of single-stranded RNA complementary to SPV_2546 or to SPD_sr95 revealed noticeable free energies of the ensembles of −23.05 kcal mol^−1^ and −26.31 kcal mol^−1^, respectively (Figure 3). This finding raises the question of whether this ORF may represent a novel example of a dual-function or bifunctional (both coding and noncoding functions) sRNA (for reviews see [142,143] or another example of a small protein possibly involved in *S. pneumoniae* physiology [144]). Of note, with the remarkable exception of having ≥95% identity to predicted small genes located at an equivalent position in the genomes of other streptococci of the Mitis group (SMG) harboring *lytA*-like genes (*lytA*_SMG_), SPV_2546 does not show any significant similarity to other previously reported proteins. Remarkably, no hits were found when SPV_2546 was searched against a global microbial sORFs catalog (GMSC) that contains more than 950 million non-redundant sORFs [145].

- The *lytA* gene, encoding the major autolysin of *S. pneumoniae*, is part of a pathogenicity island alongside the *ply* gene and is co-transcribed with *cinA*, *recA*, and *dinF*. It is regulated by multiple promoters, including a competence-specific one activated by ComX. Transcription may also be influenced by upstream elements and small RNAs, such as SPD_sr95, and possibly by a small open reading frame (*rio88*/SPV_2546) located in the *lytA* promoter region. This sORF may encode a conserved 37-amino acid peptide found across pneumococcal strains and may have both coding and regulatory functions. Its role in LytA regulation and pneumococcal physiology remains to be clarified.

## 4. Structure and Function of the NAM-Amidase

### 4.1. LytA Requires Choline-Containing Cell Walls for Activity

The *lytA* gene encodes a 318-amino acid (aa) protein with a predicted relative molecular mass of approximately 36,500. The primary translation product of *lytA* is a monomeric, low-activity form (E-form) of the enzyme. This E-form can be activated, in vitro and in vivo, into the fully active form (C-form) through a process originally termed “conversion”. In vitro, this process requires incubation at 0 °C, either with phosphocholine (P-Cho)-containing pneumococcal cell walls [146,147], 2% choline chloride [148,149], or tertiary amines like diethylaminoethanol (DEAE) [150]. *Streptococcus pneumoniae* is an auxotroph for the aminoalcohol choline [151], which decorates the cell wall teichoic acid (WTA) and membrane-anchored lipoteichoic acid (LTA) as P-Cho moieties [152,153]. Both polymers possess identical chain structures within their repeating units, indicating they are synthesized through the same biosynthetic pathway [154,155]. The repeating units contain the rare amino sugar 2-acetamido-4-amino-2,4,6-trideoxygalactose, glucose, ribitol-phosphate, and two *N*-acetylgalactosamine residues, each carrying a P-Cho moiety. The number of P-Cho residues per repeat (1 or 2) is strain-specific. WTA is linked to PG through a phosphodiester bond to the *O*6 of some *N*-acetylmuramic acid (MurNAc) residues, while the LTA chain is β-1,3 glycosidically anchored to the cell membrane via a diacylglycerol-containing lipid anchor.

### 4.2. Peculiarities of Cell Wall Degradation In Vitro an In Vivo

Pneumococcal cell walls in which the normal P-Cho component of WTA is replaced with phosphoethanolamine are unable to bind LytA and are completely resistant to autolytic degradation [148]. However, soluble teichoic acids (TAs) containing either choline or ethanolamine, prepared by treating pneumococcal cell walls with the *N*-acetylmuramidase M-1 from *Streptomyces globisporus* or with hydrofluoric extraction, were hydrolyzed by LytA to the same extent. Additionally, free choline concentrations that completely inhibited the digestion of pneumococcal cell walls both in vivo and in vitro had no effect when soluble substrates were used [156,157]. This result suggests that the strict dependence of LytA on P-Cho residues for hydrolyzing insoluble substrates, such as cell walls, is lost when acting on soluble substrates. However, it has been reported that the addition of chloroform to an actively growing *Escherichia coli* strain expressing a cloned *lytA* gene led to rapid lysis of the culture. This finding was entirely unexpected, as it represents the only known instance in which LytA has been capable of hydrolyzing the *E. coli* murein, despite the absence of P-Cho residues or TAs [158].

Another unique feature of the enzymatic activity of LytA has been reported [159]. When pneumococci were labeled in vivo with radioactive choline and allowed to undergo autolysis following the addition of PEN, the soluble products released differed from those obtained by treating radioactively labeled *S. pneumoniae* cell walls with the purified NAM-amidase. It was proposed that the in vivo-triggered amidase activity initially targets amide bonds in strategically located (or unprotected) stem peptides, which hold together large segments of the cell wall. These findings suggest that the in vivo activity of the pneumococcal autolysin is influenced by topographic constraints [159].

### 4.3. Funtional Domains of LytA and Three-Dimensional Structure

**Figure 4:**
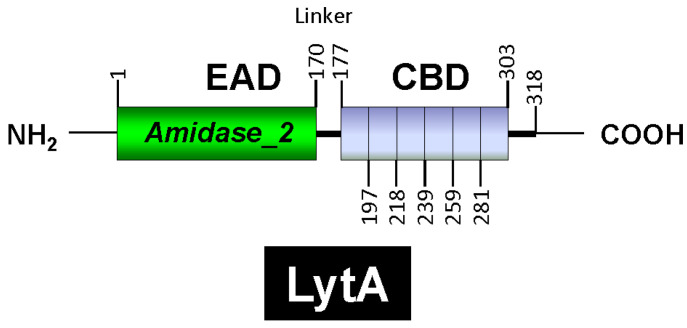
Diagrammatic representation of the modular organization of the LytA NAM-amidase. The enzymatic active domain (EAD), the linker region, and the choline-binding domains (CBD) are shown. The six choline-binding repeats are also depicted. Residue positions are indicated.

LytA is part of the amidase_2 family of proteins (which possess an *Amidase_2* domain, PF01510) [160], including Zn-dependent NAM-amidases and PG recognition proteins (PGRPs), which are highly conserved pattern-recognition molecules of the innate immune system [161]. Notably, several PGRPs (e.g., PGRP-SB1, AgPGRP-S2, AmPGRP-S1, BmPGRP-S5, DmPGRP-LB, RAjPGRP-S, and PGRP-L) also exhibit NAM-amidase activity [162,163,164,165,166]. LytA was the first identified member of the CBP family. Other CBPs, such as the autolysins LytC and CbpD, are also characterized by a choline-binding domain (CBD) responsible for binding to choline residues in WTA and LTA (for reviews, see [153,167,168,169,170,171]). In LytA, the CBD comprises the C-terminal region of the enzyme and consists of six choline-binding repeats (CBRs), each ~20 aa long, and a tail (Figure 4). The C-terminal tail deviates structurally from the other repeats. The N-terminal domain of LytA contains the active center (enzymatically active domain or EAD) (see below). Since the EAD and CBD are found in other proteins, they are often also referred to as “modules” [22].

Various approaches have demonstrated the existence and structure of the two LytA modules: sequence alignments [172,173], cloning and expression of the *lytA* gene [59,60], construction of enzymatically active chimeric proteins [174,175,176], independent expression of domains [177], and C-terminal truncations of LytA [178]. Moreover, physicochemical analyses have provided essential insights into the fine structure and organization of the LytA modules [179,180,181]. These studies revealed that at least four repeats are required for efficient autolysin anchoring to cell wall choline residues and that the active NAM-amidase forms a dimer in solution. It is known that most C-terminal residues play a crucial role in dimerization. Protein dimerization is a key factor in the regulation of proteins such as enzymes, ion channels, receptors, and transcription factors and plays a crucial role in regulating various biological processes, including enzymatic activation, signal transduction, and even pathogenic mechanisms [182,183].

An interesting characteristic of LytA is the discovery that an in-frame 6-bp deletion (ACAGGC), located between nucleotide positions 868 and 873 and encoding Thr290–Gly291, is responsible for the inability of pneumococci to undergo lysis upon the addition of Doc [184]. This deletion does not significantly compromise the enzymatic activity of the NAM-amidase under normal conditions but results in a 30% reduction in enzymatic activity in the presence of Doc. The 6-bp deletion is a distinguishing feature of certain SMG isolates, primarily *Streptococcus pseudopneumoniae*, and is responsible for the resistance of this species to lysis in the presence of Doc [184,185,186].

**Figure 5:**
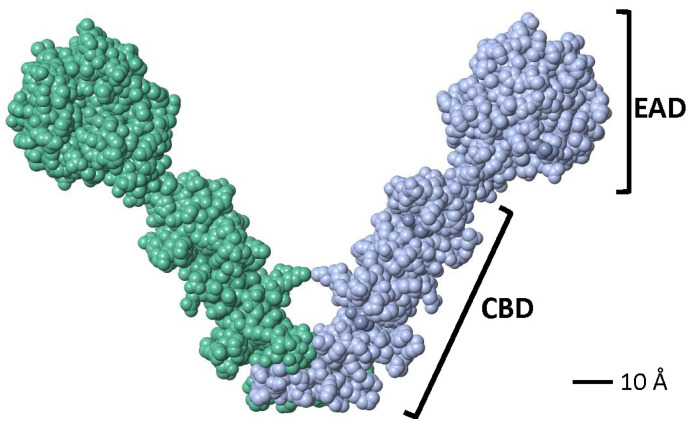
Space-filling model of the active dimer of the LytA NAM-amidase of *S. pneumoniae* (Accession number 4X36) visualized using FISTGLANCE Version 4.31 in JMOL software (http://www.bioinformatics.org/firstglance/fgij/ (accessed on 5 March 2025)). Monomers are represented in different color. EAD, N-terminal, enzymatically active domain; CBD, C-terminal, choline-binding domain.

Crystallographic studies showed that a partial LytA CBD (residues 189–318) forms a boomerang-like homodimer. The tertiary structure of each monomer comprises independent β-hairpins and a connecting loop arranged in a left-handed superhelix, forming a solenoid fold [187,188]. Each pair of consecutive CBRs forms a canonical choline-binding site (CBS) containing two hydrophobic layers. Aromatic residues from the hairpins and a hydrophobic residue (Met or Leu) from the connecting loop create the CBS. The three-dimensional structure of a recombinant LytA EAD (residues 1–172, with Cys60 and Cys136 replaced by Ala) was later elucidated [189]. The EAD forms an elliptical globular domain with a Zn^2+^ ion coordinated by His26, His133, and Asp149. Site-directed mutagenesis revealed that Glu87 and His147 are key active site residues. Consistent with earlier findings [147], enzymatic activity is inhibited by 1–10 mM ZnCl_2_ in vitro [189]. Later studies reported the complete three-dimensional structure of the LytA NAM-amidase dimer [190], where the subunits form a boomerang-like structure with an internal angle of 85° and arms 106 Å long (Figure 5). Residues 1–170 constitute the EAD, and residues 177–318 (including the tail) form the CBD. Both domains are connected by a six-residue linker. The CBD contains six CBSs, including five canonical and one single-layered site. The consensus sequence for CBRs, GWXKX_4_–_5_WYYφX_3_–_5_GXMX_2_–_3_ (where φ represents a hydrophobic residue), aligns well with prior studies on other pneumococcal CBPs [191].

- LytA, the main autolysin of *Streptococcus pneumoniae*, is synthesized as an inactive form that transforms an active enzyme in the presence of choline-containing compounds. LytA specifically binds to P-Cho on WTAs and is essential for cell wall degradation. It has two domains: a Zn^2+^-dependent enzymatic domain (EAD) and a choline-binding domain (CBD) with six repeats, crucial for anchoring and dimerization. Structural studies show LytA forms a boomerang-shaped dimer.

## 5. Regulation and Control of LytA

Generally speaking, several mechanisms are employed to ensure that autolysins do not compromise the structural integrity of the cell [192,193]. These mechanisms include regulating PG hydrolase levels through (1) transcriptional or (2) post-transcriptional control, (3) direct activation or inhibition by regulatory proteins or small molecules, and (4) spatial regulation via proteins, surface polymers, or modifications of the PG substrate, allowing cell wall enzyme activity to be restricted to specific cellular sites. Remarkably, in virtually all cases, the regulation of autolytic enzymes involves a combination of control mechanisms, allowing for precise tuning of activity as well as spatial and temporal regulation.

In *S. pneumoniae*, the access of LytA and certain other CBPs to the cell wall requires interaction with and translocation across the cell membrane, because of the lack of a signal peptide [194]. Early results have shown that LytA is located in the cellular envelope of *S*. *pneumoniae* and *E. coli* through immunocytochemical labeling of ultrathin sections and whole-mounted cells [195]. In whole *S. pneumoniae* cells, it has been observed that the labeling is mainly found in the septal region. In addition to electron micrographs, cell fractionation studies in *E. coli* confirmed that the pneumococcal amidase is peripherally localized and weakly associated with the outer surface of the cytoplasmic membrane. This interaction is independent of choline and, notably, the NAM-amidase remains unprocessed during translocation [195]. Interestingly, recent studies have demonstrated that the LytA peptide 239-TGWKKIADKWYYFN-252, a segment of CBR4 [190], can reversibly change from a β-hairpin, in aqueous solution, to a well-defined, stable α-helix through its interaction with dodecylphosphocholine (DPC) micelles but not with individual phosphocholine molecules [196,197]. Additionally, it has been reported that the aromatic side chain of Y250 is involved in a stronger interaction with DPC micelles than Y249 [198]. This mechanism may represent a general strategy for sorting some proteins to the bacterial surface to perform their physiological functions. These proteins and peptides that change in folding have been referred to by various names, including chameleon/metamorphic proteins, proteins with two folds, switch peptides, and turncoat polypeptides (for reviews, see [199,200,201,202]).

### 5.1. Transcriptional Regulation

The vast majority of bacteria encounter physical and chemical changes in their environment that could be sensed as stress [203,204,205]. An obvious one is temperature. The surface temperature of the anterior nares is approximately 30 °C to 32 °C at the end of inspiration, increasing to about 34 °C in the posterior nasopharynx and tonsillar region [206,207]. These mucosal surface locations are notably cooler than the core body temperature of 37 °C, which is where bacteria replicate during IPD. Recent results indicate that transcriptional upregulation of *dinF* and *cinA* (but not *lytA*) occurs at 34 °C compared to higher incubation temperatures (37 °C or 40 °C) [208]. On the other hand, gene expression analysis of eleven targets demonstrated that *lytA*, *lytC*, *comD*, and *pavA* were the most highly expressed pneumococcal genes in the nasopharynx of healthy children, while the others (*ply*, *codY*, *mgrA*, *nanA*, *nanB*, *pspA*, and *rrgB*) exhibited only moderate to low expression levels [209]. An increase in temperature from 37 °C to 40 °C has been shown to significantly accelerate pneumococcal autolysis rates [210]. Additionally, heat stress—defined as transient exposure of pneumococci to 42 °C—induces both early and late competence genes (presumably including *lytA*) in a time- and dose-dependent manner [211]. This thermal regulation depends on the HtrA chaperone/protease and its proteolytic activity. HtrA (high-temperature requirement A) is a component of the CiaRH regulon and is recognized as an important virulence factor [212]. On the other hand, an in vitro study found no evidence of RNA thermosensors regulating the transcription of *lytA* [213]. Using a human middle ear epithelial cell line, it was observed that *lytA* transcription was significantly induced exclusively in pneumococci attached to the epithelium under simulated pathological middle ear mucosa conditions [214].

Interestingly, competence development is induced in *S. pneumoniae* under lethal stress conditions, including antibiotic treatment (for recent reviews, see [215,216]). Several hypotheses have been proposed regarding the role of competence in transformation, including serving as a source of nucleotide components, enabling DNA acquisition for genome repair, or facilitating the uptake of novel genetic material to drive evolution [217]. However, it is now widely accepted that competence, though not necessarily transformation, provides protection against both DNA-damaging and non-DNA-damaging stresses [218,219,220].

The fact that *lytA* is part of the same operon as *cinA* (see Section 3) strongly suggests that competence development in *S. pneumoniae* results in increased *lytA* transcription. However, it should be noted that transcript levels do not necessarily correlate with protein biosynthesis [221,222]. It has been well-documented that the incubation of pneumococci under conditions favorable for competence development leads to increased chromosomal DNA release into the medium and accelerated autolysis during the stationary phase, primarily due to LytA and LytC [77,223]. One study reported that *lytA* expression increases approximately four-fold in pneumococcal cells undergoing genetic transformation [224]. More recent data demonstrated that 10 min after the addition of the competence-stimulating peptide (CSP)—a 17-residue extracellular peptide that is ribosomally synthesized as a precursor peptide (ComC) [225,226]—*lytA* transcription increased up to 10-fold before rapidly returning to near-normal levels within another 10 min [135,227].

Aprianto and colleagues had observed that when *S. pneumoniae* D39 was incubated with human type II lung epithelial cell line A549, *lytA* transcription (along with that of many other competence-related genes) increased beginning 60 min after infection and remained elevated throughout the experiment [228]. Activation of *lytA* transcription during in vitro competence induction has been corroborated by recent independent studies [229,230]. Additionally, CSP1-E1A (a CSP1 analog) was able to competitively inhibit the development of competence and reduced the expression of pneumococcal virulence factors like CbpD and LytA in vitro [231]. In addition, overexpression of the *S. pneumoniae spxA1* gene represses transcription of the early competence operon *comCDE*, thereby inhibiting the onset of competence [232]. Subsequently, it was noted that the deletion of *spxA1* led to earlier autolysis in the stationary phase, although no significant impact was observed during logarithmic growth [233]. The *spxA1* gene forms an operon with *tenA*, which slightly overlaps at its 3′ end. Together, these two genes represent a novel example of a type II toxin–antitoxin system in pneumococci [234].

Under in vitro conditions, the competence regulon in pneumococci governs both genetic transformation and virulence. However, detailed investigations of competence induction during host infection have only recently been undertaken [235,236]. Strain D39 and several other clinical isolates were used to study competence development in a mouse model of pneumonia-derived sepsis. Notably, in contrast to the characteristic short transient burst of competence observed in vitro, the competent state during pneumonia-derived sepsis was prolonged and persistent. Competence began approximately 20 h post-infection, facilitating systemic invasion, and sepsis development. Notably, the pneumococcal inoculum concentration did not significantly impact competence induction kinetics. Interestingly, exogenously added CSP failed to modulate the onset kinetics of competence development in vivo [235]. Proteomic analyses have shown that activation of competence is a key feature of pneumococcal meningitis progression. In a mouse model of infection, the absence of ComDE and the corresponding inhibition of competence development (see Section 6) resulted in diminished meningeal inflammation and milder disease symptoms compared to infections with wild-type pneumococci [237]. Using a zebrafish larval meningitis infection model, it was found that *lytA* transcription was lower when the larvae were infected with a Ply^+^ D39 strain compared to infection with a Ply^−^ mutant; the reasons for this discrepancy remain unclear [238].

Furthermore, under in vivo conditions, *lytA* transcription was slightly (but significantly) upregulated in the heart compared to the nasopharynx, lungs, kidneys, or blood when the TIGR4 strain was used [239,240,241]. Across all these studies, strain-dependent variability was evident, indicating that different pneumococcal strains exhibit diverse transcriptomic profiles within the same organ and across different infection sites. Furthermore, analysis of RNA from pneumococci isolated from infected rabbit blood, cerebrospinal fluid (CSF), or bacteria attached to a pharyngeal epithelial cell line in vitro revealed decreased expression of *lytA* only in the CSF [242]. Autolysis dependent on LuxS was suppressed in a *luxS* mutant, indicating that LuxS (encoding S-ribosylhomocysteine lyase) is somehow involved in the control of LytA-dependent autolysis [243]. Adding 0.4% BSA to the medium further protected *luxS* mutants from autolysis. Surprisingly, these findings contrast with results showing that PEN (0.5 × minimum inhibitory concentration, MIC) treatment of strain D39 upregulated genes in both the CiaRH operon and *luxS* [244]. Notably, biofilms formed by a Δ*luxS* mutant showed unchanged *lytA* transcription levels when compared to the wild-type D39 strain, in a middle ear rat infection model [245]. This result contrasts with a previous report showing that LuxS regulates the transcript levels of *lytA* during in vitro biofilm formation [246]. The *luxS* gene was upregulated by 3.4-fold in the presence of sand dust—a common air pollutant of arid and semi-arid regions of many countries that is a risk factor for otitis media—and similarly, the *lytA* gene was upregulated by 2.3-fold in the presence of sand dust [247]. Upregulation of *lytA* transcription was also observed when *S. pneumoniae* TIGR4 strain was exposed for 2 h to nicotine-containing electronic cigarette vapor extract [248].

Remarkably, and as determined by Western blotting, it has been reported that some macrolide antibiotics, i.e., azithromycin and erythromycin, inhibit the release of LytA into the supernatant of cultures obtained until the stationary phase was reached [249]. Additionally, both macrolides significantly downregulate the transcription of the *ply* gene, while *lytA* transcription remains unaffected [249]. A follow-up study confirmed the dual effect of erythromycin on inhibiting both Ply synthesis and release, whereas clarithromycin significantly suppressed *ply* transcription but upregulated *lytA* transcription, leading to enhanced autolysis [250].

Additionally, pneumococcal autolysis and fratricide can be modulated by interactions with other bacterial species within a polymicrobial community. When *S. pneumoniae* TIGR4 was co-cultured with nontypeable *H. influenzae*, the expression of *lytA* (and *cbpD*) was downregulated, resulting in reduced LytA production [251,252].

### 5.2. Post-Transcriptional Regulation

#### 5.2.1. Two-Component Systems and LytA

Bacteria adapt to environmental changes through a mechanism known as the two-component regulatory system (TCS) (also referred to as the “two-component signal transduction system” or “two-component system”) [253]. A TCS typically consists of at least two proteins: a response regulator and its corresponding sensor histidine kinase.

*Streptococcus pneumoniae* encodes 13 TCSs and a single orphan response regulator [254,255]. Among these, the CiaRH (competence induction and altered cefotaxime susceptibility) system (TCS05) regulates various processes, including autolysis. Increased autolysis of *ciaR* mutant cells has been observed under several conditions: upon addition of CSP (see above), during the stationary growth phase, when triggered by choline depletion, or following treatment with early or late inhibitors of cell wall biosynthesis [256]. Zähner et al. noted that the phenotype of a strain is influenced by individual *ciaH* mutations [257]. Mutants with an activated CiaRH system (designated *cia* ON), such as strain RCH1 with the *ciaH*_C306_ mutation (Thr230 to Pro) or strains harboring mosaic or point mutations in *pbp2X* combined with the *ciaH*_C103_ allele, are resistant to lysis induced by a variety of early and late cell wall inhibitors and are also less susceptible to drugs such as cycloserine, bacitracin, and vancomycin (VAN) [256]. In contrast, loss-of-function CiaRH mutants are hypersensitive to these drugs and lyse rapidly at the stationary growth phase. CiaR directly regulates 15 promoters that drive the transcription of 24 genes organized into five operons and ten monocistronic units [258,259]. Among these, five monocistronic units encode noncoding RNAs (csRNAs, or CiaR-dependent sRNAs). These csRNAs (csRNAs1–5), also known as CcnA–E (CiaR-controlled noncoding RNAs) [260], play a critical role. Specifically, csRNA4 and csRNA5 regulate autolysis during the stationary phase [258]. If both RNAs are absent, autolysis initiates significantly earlier and proceeds faster than in wild-type strains. These sRNAs lack any obvious complementarity to *lytA* mRNA, suggesting that their effects on autolysis are unlikely to involve direct interference with LytA biosynthesis [258].

Similarly to CiaRH, the LiaFSR system (TCS03) is activated during cell wall stress caused by the activity of the PG hydrolases CbpD, LytA, or LytC [254]. Like CiaRH, LiaFSR is not essential for survival. However, the LiaFSR system appears critical for protecting competent pneumococci from the potentially lethal effects of fratricide (see Section 2). In a Δ*comM* mutant, CbpD activates the LiaFSR system in conjunction with LytA and LytC. Additionally, two members of the LiaFSR regulon, *spr0810* and *spr0351* (CbpF; formerly known as CbpC) [117,171], or PcpC [170,261,262]), are crucial for inhibiting fratricide-associated cell lysis [263]. Without a functional LiaFSR system, cell lysis doubles in both ComM-proficient and -deficient cells. CbpF blocks LytC-induced autolysis at 30 °C in vitro, potentially by preventing the access of the lysozyme to its substrate [264].

TCS09 remains poorly understood, with significant strain-specific functional differences [265]. When *S. pneumoniae* D39 was treated with Triton X-100, TCS09-deficient mutants exhibited higher autolysis rates than isogenic parental strains [266]. The strain-specific effects of TCS09 on cellular processes remain unclear, though TCS09 is known to regulate carbohydrate metabolism and, likely indirectly, influence the amount of capsular polysaccharide (see Section 5.2.3) [267].

#### 5.2.2. Other Mechanisms of Regulation

Exposure to serum stimulated the expression of the pneumococcal lipase LipA at both the mRNA and protein levels. In the presence of serum, the Δ*lipA* mutant exhibited accelerated lysis rates and elevated LytA expression compared to the *lipA+* parental strain, both in vitro and in vivo. Moreover, it was found that the expression of *lytA* in a sepsis model was inhibited in the D39 *lipA^+^* strain, but not in the Δ*lipA* mutant and that the induction of *lipA* expression results in the inhibition of autolysis [268].

The incorporation of d-alanine in LTAs is accomplished in a two-step reaction involving d-alanine-d-alanyl carrier protein ligase (DltA) and d-alanyl carrier protein (DltC). During the stationary phase, autolysis began earlier in R6 Δ*dltA* and D39 Δ*dltA*, while it remained unaffected in strain Rx (another D39 derivative) [269]. In other bacteria, the absence of d-Ala in LTA increases the net negative charge of cell walls, inducing autolysis [270,271,272].

Although the polyamine spermidine is dispensable for growth in vitro, it plays a crucial role in regulating LytA activity, likely through interactions with negatively charged molecules such as TAs [273].

#### 5.2.3. Antibiotic Tolerance

Antibiotic tolerance is defined as the ability of bacteria to survive transient exposure to bactericidal antibiotics, even at concentrations far exceeding the minimum inhibitory concentration (MIC) [274,275]. Unlike resistance, tolerance applies exclusively to bactericidal antibiotics and not to bacteriostatic ones, as all bacteria are expected to survive transient exposure to bacteriostatic antibiotics, which merely arrest growth rather than kill. In contrast to resistance and tolerance—attributes of entire bacterial populations—“persistence” refers to the ability of a subpopulation of bacteria to survive high antibiotic concentrations [276]. Confusion between the concepts of tolerance and persistence remains common [277].

While antimicrobial resistance has been extensively studied [278], the molecular mechanisms underlying antibiotic tolerance are less well understood, particularly in *S. pneumoniae* [279]. These mechanisms may also vary depending on the specific antibiotic used [280]. As previously noted, LytA-deficient *S. pneumoniae* strains exhibit tolerance when treated with antibiotics that inhibit cell wall synthesis [51,57]. Moreover, environmental factors, such as the pH of the growth medium, influence bacterial lysis [281,282]. These early studies demonstrated that the bacteriolytic effect of β-lactam antibiotics on *S. pneumoniae* depended on pH; lysis was inhibited when the pH of pneumococcal cultures remained below 6.0 during PEN treatment. Drug-treated cells merely ceased growth, with a significant reduction in cell death. Additionally, this effect was reversible, as lysis and loss of viability could be induced by post-incubating drug-treated bacteria at a lysis-permissive pH [281].

To date, only two studies from Iran have reported the isolation of VAN-resistant pneumococci (MIC 2–16 μg mL^−1^) [283,284]. However, the current MIC breakpoint for VAN in *S. pneumoniae* is 2 μg mL^−1^ [285] and it is generally accepted that *S. pneumoniae* is universally susceptible to VAN [286]. Nevertheless, a recent study [287] described an invasive serotype 4 strain with reduced VAN susceptibility (MIC 1 μg mL^−1^ compared to the typical 0.38–0.5 μg mL^−1^ for susceptible pneumococci), harboring a *vanG*-type resistance element [288,289]. Additionally, several studies have reported the isolation and characterization of VAN-tolerant pneumococci (for reviews on early studies, see [290,291]). This raises concerns as antibiotic tolerance may facilitate the evolution of resistance [292,293].

The simplest explanation for antibiotic tolerance is the failure of the bacterium to express an enzymatically active LytA autolysin. Although this feature is widely recognized, it is important to emphasize that, with the notable exception of a specific group of clonal pneumococci—naturally occurring only in horses—which possess a chromosomal deletion leading to a pneumolysin–autolysin fusion gene [113,294,295], to the best of my knowledge, only one clinical isolate of pneumococcus has been demonstrated to be a true *lytA* mutant [296,297]. Table 1 summarizes studies dealing with the molecular basis of antibiotic tolerance in *S. pneumoniae*.

Capsular polysaccharide (CPS), the primary virulence factor of *S. pneumoniae*, also negatively influences lysis efficiency. Encapsulated strains from different serotypes demonstrate reduced lysis upon PEN or VAN treatment compared to nonencapsulated mutants, though some serotype-specific differences in lysis have been noted [320]. It is conjectured that the capsule may inhibit LytA from accessing their target structure, PG, or may slow down the translocation of LytA to the cell wall, although the possibility that the capsule itself may protect from osmolysis cannot be completely discarded. This link between capsule presence and increased antibiotic tolerance was confirmed independently [297]. Furthermore, nonencapsulated D39 mutants underwent lysis more rapidly and exhibited increased susceptibility to Triton X-100-induced autolysis compared to their encapsulated counterparts [266]. More recently, additional evidence has confirmed that the capsule protects pneumococci from LytA-induced lysis [321].

Analysis of clinical VAN-tolerant isolates revealed additional insights. For example, strain S3 of serotype 23F exhibited VAN tolerance due to a LytA NAM-amidase deficiency caused by a frameshift mutation in the *lytA*_S3_ gene [297]. In addition, sequencing of the *ciaRH* genes in the Tupelo strain revealed a mixed population in the Tupelo stock, containing a mutation in the *ciaH* gene [297]. Only the mutants with a GCC-to-TCC mutation at position 592 from the start codon (ATG) of *ciaH* exhibited VAN tolerance. Additionally, exponentially growing Tupelo cells displayed a reduced LytA autolysin synthesis rate (~35% lower) compared to strains R6 and TIGR4.

Despite extensive research, misconceptions about VAN tolerance, persistence, and resistance continue. For instance, while the VncRS system is unrelated to VAN resistance, some studies have mistakenly linked it to this phenotype [322,323]. This underscores the ongoing challenges in fully understanding VAN tolerance in *S. pneumoniae*.

### 5.3. Regulatory Molecules

#### 5.3.1. LTA

Although extensive studies have been conducted, it has been traditionally believed that the autolytic activity of LytA is regulated at the post-translational level by the membrane-anchored LTA and strictly requires the presence of P-Cho in WTA for enzymatic activity (see above). When pneumococcal LTA was added to growing pneumococci, it induced chain formation, prevented culture lysis during the stationary growth phase, and inhibited lysis caused by PEN or VAN. However, this inhibition could be reversed with low concentrations (0.2%) of Doc. Notably, WTA remained inactive even at concentrations several hundred-fold higher [324,325]. Furthermore, mere binding to LTA is unlikely to be responsible for the inhibitory effect; rather, the inhibition likely arises from the inaccessibility of the substrate to the NAM-amidase when bound to micellar LTA. Actually, LTAs that had its lipid moiety removed through lipase digestion lost its ability to inhibit the amidase, correlating with its reduced capacity to form micelles [149].

Pneumococci control LTA levels by modulating the abundance of the LTA synthase TacL [133,326] (previously referred to as RafX [327]). TacL depletion during growth in liquid media leads to premature LytA-dependent autolysis during the exponential growth phase. During this phase, *S. pneumoniae* primarily synthesizes LTAs that bind and sequester the major autolysin LytA. Additionally, the observed increase in WTAs when LTA synthesis is blocked suggests that the two pathways are antagonistic and likely compete for a shared precursor, i.e., a polymer that is linked to an undecaprenyl phosphate lipid carrier. By controlling the TacL levels, the cell can regulate the flux into either LTA or WTA synthesis, given their reliance on the same precursor [133,326]. Elevated LTA levels during exponential growth sequester the CBD of LytA away from the cell wall, thereby reducing its hydrolytic activity during this phase. However, during the stationary phase or in response to cell wall-targeting antibiotics (e.g., PEN), TacL levels decrease by the membrane protease FtsH leading to reduced LTAs and increased WTAs. Furthermore, consistent with early findings [328,329], LTAs are released from cells during autolysis. Coupled with the shift in TA synthesis favoring WTA over LTA, this release allows for rapid LTA depletion and the re-localization of LytA to WTAs, where it facilitates PG hydrolysis [133]. A recent report indicated that ComE, a transcription factor essential for competence development [330], negatively regulates the transcription of *tacL* hampering pneumococcal transformation [331]. This highlights a connection between competence development and the regulation of TA synthesis.

#### 5.3.2. Enzymatic Activation of LytA

As already mentioned, the E-form (low activity) of LytA exists as a monomeric protein, whereas the active C-form is a dimer, shaped through the tail-to-tail association of two monomers in the presence of choline [182,332]. Once activated, LytA cannot revert to its low-activity form by dialysis against a choline-free buffer. In fact, the complete removal of choline leads to the irreversible denaturation of the enzyme. Interestingly, the temperate phages φB6 and φHER of *Streptococcus mitis* encode two LytA-like NAM-amidases, designated LytA_B6_ and LytA_HER_, respectively [333]. These enzymes are 318 residues long, sharing a global > 83% identity (>90% similarity) with the pneumococcal LytA (LytA*_Spn_*). Similarly to LytA*_Spn_*, the phage-derived lytic endolysins also require the activation for full enzymatic activity, which involves a transition from a monomeric to a dimeric state. However, unlike pneumococcal NAM-amidase, the active phage endolysins can reversibly deactivate when choline is removed from the solution, causing the proteins to adopt a predominantly monomeric structure [333]. Sequence alignment of LytA with the two phage NAM-amidases revealed a key distinction between the pneumococcal and *S. mitis* phage NAM-amidases: at position 317, LytA*_Spn_* features a Val residue, whereas the phage enzymes possess a Thr residue. Structural studies of LytA*_Spn_* indicated that Val317 interacts with Phe283 and Tyr294 within the same monomer to form a hydrophobic core critical for maintaining the dimeric structure [188]. Like the pneumococcal LytA, the isolated CBD moieties of the temperate phage enzymes described undergo a reversible dimer↔monomer transition triggered by the addition or removal of choline, respectively. The activation of the E-form to the C-form in these enzymes is invariably linked to these monomer–dimer transitions. However, it remains uncertain whether dimerization and activation are simply concurrent processes or if dimerization contributes, at least partially, to the activation effect.

**Figure 6:**
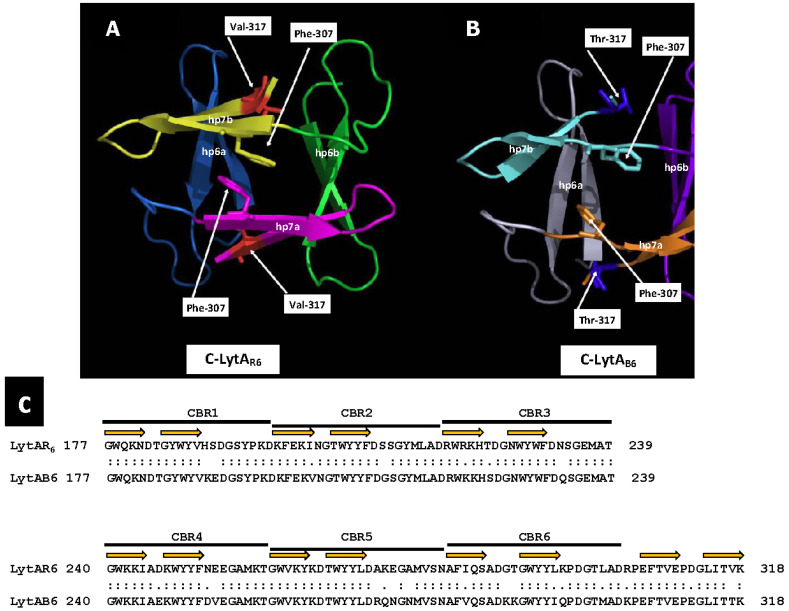
Dimerization interface of C-LytA_R6_ and C-LytA_B6_. Ribbon diagrams showing the dimerization interface of the C-termini of LytA_R6_ (from LytA*_Spn_*) (**A**) and C-LytA_B6_ (from LytA_B6_, a PPH endolysin) (**B**). Monomers are labeled *a* and *b*. Each hairpin (hp) is colored differently. The side chains of Phe307 and Val/Thr317 are shown. An alignment of the CBD of both enzymes is shown in panel (**C**). The portions of the sequences that form the first and second strands of the hairpin are indicated by orange arrows.

In a preliminary study, our group determined the three-dimensional structures of the CBD of LytA_B6_ (C-LytA_B6_) (WP_000350519) using X-ray crystallography and ^13^C and ^15^N NMR spectroscopy to analyze the dimeric and monomeric states in the presence and absence of choline, respectively. Our findings revealed that the three-dimensional structure of the C-LytA_B6_ dimer closely resembles that of C-LytA_R6_, as expected, given their high sequence similarity (84% identity, 92% similarity over a 142 aa overlap) (Figure 6). However, in the absence of choline, part of the C-LytA_B6_ structure undergoes significant rearrangements, particularly in CBR5 (aa positions 260–281), resulting in notable architectural distortion. In addition, Thr317 does not integrate into the hydrophobic core due to its hydrophilic nature. In the absence of choline, the hydrophobic core in C-LytA_B6_ is expected to disassemble significantly faster than in C-LytA_R6_, allowing C-LytA_B6_ to more easily adopt its stable conformation without choline. Conversely, transitioning from the choline-free to the choline-bound form requires reassembly of the hydrophobic core. Temperatures near 273 K may facilitate this rearrangement by weakening hydrophobic interactions, though such conditions may not sufficiently lower the energy barrier for the disassembly of the tightly packed hydrophobic core in C-LytA_R6_. Furthermore, when LytA transitions from the low-activity to the active form, the catalytic unit shifts from having a single catalytic site to possessing two, substantially increasing the autolysin’s catalytic efficiency. More importantly, the transition from the E-form to the C-form exposes additional cell wall binding sites per monomer and forms a new catalytic dimer containing twelve CBSs. This significantly enhances the affinity of the catalytic domain for the cell wall, resulting in increased catalytic efficiency. This improved affinity arises from the additive free energy of binding provided by the linked CBSs and the reduced entropy cost due to their linkage. Such mechanisms suggest that linking binding fragments with millimolar affinities can lead to compounds exhibiting subnanomolar affinities [334]. In addition, among twenty tested LytA_R6_ mutants created by site-directed mutagenesis only LytA_Y294L_, LytA_V317W_, and LytA_L314T_ produced an enzyme that remained fully active but could, unlike the wild-type enzyme, reversibly return to its low-activity E-form upon dialysis [335]. However, despite these results, it is not clear if dimerization and activation are mere parallel phenomena or if dimerization can explain, at least in part, the activation effect. Additional insights from ^1^H NMR spectroscopy and analytical ultracentrifugation of the low-activity form of LytA_B6_ revealed that incubation with 140 mM choline chloride at 37 °C for 5 min induces self-association. However, full activation of LytA_B6_ was achieved only after incubation with its ligand at 0 °C [335].

It is conceivable that beyond the self-association involving the CBD of LytA, structural modifications at the EAD may also contribute to its activation. Previous studies have shown that the NAM-amidases AmiB and AmiC from *E. coli* have their active sites blocked by an α-helix, with enzyme activation occurring upon displacement of this occluding helix. Further research demonstrated that EnvC specifically activates AmiB, while NlpD activates AmiC (reviewed in [192]). Additionally, AmpD from *Citrobacter freundii*, a member of the amidase_2 protein family like LytA, also adopts an inactive (“closed”) conformation that can transition to an active (“open”) state [336]. However, the triggering event for this conformational shift in AmpD remains unknown. In addition, a recent study revealed that LytB of *S. pneumoniae*, a CBP with *N*-acetylglucosaminidase activity (EC 3.2.1.96) responsible for the final step of daughter cell separation [337,338,339], also exhibits both inactive/closed and active/open conformations at its catalytic module [340]. In its closed conformation, access to the active site is blocked, whereas in the open conformation, the substrate-binding cavity is exposed. It has been suggested that this transition may occur through the accommodation of PG chains within the catalytic module [340]. A similar mechanism may underlie the enzymatic activation of LytA.

### 5.4. Spatial Regulation

The data outlined above suggest that LytA activity may be influenced by the structure of the PG network, including factors such as MurNAc *O*-acetylation, the presence or absence of branched muropeptides, and/or the spatial organization of WTAs. Multiple independent studies employing various experimental approaches have demonstrated that LytA localizes at the cell division septum [195,341,342,343], which corresponds to the site of the newly synthesized cell wall during cell division [344,345]. In the exponential phase, LytA is either diffusely distributed in the cytoplasm or attached to the membrane [149,343]. During the lytic phase, LytA binds to the surface of neighboring non-lysed cells specifically at the mid-cell position [343].

#### Capsular Polysaccharide, WTA and Autolysis Control

Recent experimental evidence indicates that several genes involved in capsule biosynthesis also contribute to regulating the NAM amidase LytA. In *S. pneumoniae*, capsular genes are organized into a single operon (*cap*/*cps*), with only the first four genes (*cap*/*cpsABCD*, now renamed *wzg*, *wzh*, *wzd*, and *wze*) being conserved across all pneumococcal serotypes, except for types 3 and 37 [194,346,347,348]. The first gene in the *cps* operon (capA/*cpsA/wzg*) encodes a protein from the LCP (LytR–CpsA–Psr) family, which typically facilitates the attachment of cell wall glycopolymers to the PG backbone of Gram-positive bacteria via a phosphodiester linkage [349,350]. Wzd and Wze co-localize at the division septum and bind Wzg [321]. These and other proteins form part of the pneumococcal divisome [351,352]. Compared to the wild type, pneumococci with mutations in *wzd* or *wze* lack capsular material at the midcell (although it remains on other parts of the cell), exhibit stronger binding to LytA, are more susceptible to LytA-induced lysis than encapsulated mutants, and show reduced virulence [321]. Notably, Δ*lytR* pneumococci exhibit reduced growth, premature autolysis [353], and produce the same amount of capsular polysaccharide as *cpsA lytR* double mutants [354]. Additionally, a *psr* mutant produces a reduced amount of capsule [355]. Recent evidence has also shown that LytR is the primary enzyme responsible for mediating the final step in WTA formation and, along with ComM, plays a critical role in providing immunity against CbpD [229]. In addition, depletion of FtsZ—the leader protein of the cell division machinery [356]—in *S. pneumoniae* is lethal and results in LytA-induced autolysis and loss of viability that is independent of LytA autolysis [357].

The WhyD protein is a membrane-anchored WTA hydrolase responsible for removing WTAs in *S. pneumoniae* [358]. WhyD regulates WTA levels to prevent LytA from being mistakenly activated and causing lysis during exponential growth. Crucially, WhyD activity reduces WTA content specifically at sites of PG synthesis. Interestingly, WhyD is essential not only for controlling the overall abundance of WTAs but also for restricting their localization to the midcell, where cell wall synthesis occurs. In cells lacking WhyD, WTA levels are significantly elevated, while LTA levels remain unaffected [358].

- LytA is tightly regulated to prevent premature lysis. Its activity is controlled at multiple levels, including transcriptional and post-transcriptional regulation, enzymatic activation via dimerization, spatial localization to the division septum, and modulation by two-component systems and surface structures like teichoic acids and capsule. Environmental cues, stress, and competence development also influence LytA expression. These complex regulatory networks ensure precise control of LytA during growth, autolysis, and pathogenesis.

## 6. The LytA Autolysin as a Virulence Factor

*Streptococcus pneumoniae* is primarily a human pathogen; however, it has also been isolated from pets [359], equine species [295,360,361], and great apes [362,363,364,365,366]. It has been realized that, in most cases, these infections likely originate from human carriers. To investigate the role of LytA (and other virulence factors) in pneumococcal pathogenesis, various animal models are utilized. Mice are by far the most commonly used laboratory animals, although other models—including rats, rabbits, chinchillas, gerbils, guinea pigs, swine, and nonhuman primates—have also been employed for pathogenicity studies and to assess antibiotic and/or vaccine efficacy [367,368]. Additionally, zebrafish models, both embryos and adults, are currently being explored [369,370]. Several studies have evaluated the pathogenic potential of LytA using various animal infection models. As documented in Table 2, 12 out of 15 studies revealed that the NAM-amidase LytA is indeed a virulence factor, this is, the *lytA* pneumococcal mutants exhibited lower virulence compared to their wild-type progenitors, regardless of the strain used for infection, the route of inoculation, or the animal model employed.

### 6.1. Interactions Between LytA and Host Defenses

The complement system plays a crucial role in the immune defense against *S. pneumoniae*. To evade a complement attack, pneumococci have developed several mechanisms that inhibit complement-mediated opsonization and subsequent phagocytosis [385]. Previous studies have shown that the combined effects of β-lactam antibiotics and specific antibodies enhance bacterial clearance in cases of sepsis caused by antibiotic-resistant *S. pneumoniae* strains (for a review, see [386]). This phenomenon can be explained by the observation that the recognition of antibiotic-resistant *S. pneumoniae* strains by the complement component C3b is enhanced in the presence of specific anti-pneumococcal antibodies and subinhibitory concentrations of macrolides or β-lactams [378]. Notably, LytA has been identified as a key factor in bacterial recognition by the complement system, as phagocytosis by neutrophils and alveolar macrophages was found to be increased in *lytA* mutants [378]. Additionally, LytA has been shown to play a direct role in host immune evasion by preventing recognition by C3b [379]. LytA inhibits the activation of both the alternative and classical pathways of the complement system. The activation of complement cascades results in the formation of C3b allowing microbial opsonization and enhancing phagocytosis [387]. In addition, LytA recruits complement system down regulators (C4BP and FH) and, if enzymatically active, cleaves C3b and iC3b components bound to the pneumococcal surface [379]. These findings highlight the critical role of LytA in evading complement-mediated immunity and phagocytosis.

The P-selectin glycoprotein ligand-1 (PSGL-1) is a mucin-like transmembrane glycoprotein expressed on all leukocytes. It serves as the primary ligand for P-selectin and also interacts with E- and L-selectins, playing a crucial role in protecting against invasive bacterial infections ([388] and references therein). PSGL-1 has been shown to bind the LytA autolysin, promoting the phagocytosis of *S. pneumoniae* [389]. Studies using mouse models of pneumococcal disease have demonstrated significantly higher bacterial loads in the blood of *PSGL-1*^−/−^ mice. During pneumonia, PSGL-1 regulates the extent of pneumococcal spread from the lungs to the bloodstream, while in systemic infections, it plays a key role in bacterial clearance by controlling replication in circulation. Although *PSGL-1*^−/−^ mice exhibited increased neutrophil and macrophage counts in the blood during systemic infection, they were less effective in controlling the infection due to the absence of this functional receptor. These findings highlight the critical role of the LytA-PSGL-1 interaction in the innate immune response against *S. pneumoniae* [389].

### 6.2. LytA Cooperates in the Release of Additional Virulence Factors

In addition to the significant direct involvement of LytA in the pathogenesis of pneumococcal disease, the triggering of LytA facilitates the release of intracellularly located virulence factors. A main example is Ply, a cholesterol-dependent pore-forming toxin and one of the primary pneumococcal proteins contributing to virulence (for reviews, see [114,115,390,391,392,393]). Given that, as LytA, Ply lacks a canonical N-terminal signal peptide for export, its release has been debated, with some attributing it to LytA-dependent autolysis. While most studies emphasize the importance of autolysis in Ply release [249,250,394,395], others suggest that Ply may reside on the bacterial outer surface independently of LytA [375,396]. Additionally, Ply release has been reported to increase with *spxB* gene expression, encoding pyruvate oxidase, before the stationary phase [397]. More recently, it has been reported that inhibition of H_2_O_2_ production by three different mutants (Δ*spxB*, Δ*lctO*, and Δ*spxB*Δ*lctO*) is accompanied by a reduction in the release of Ply [398]. The involvement of an accessory Sec system (SecY2A2) in Ply export has also been proposed [399,400,401]. Furthermore, the cell wall hydrolase activity of LytA and PG cleavage may play a key role in regulating toxin sorting during secretion, as observed in *Staphylococcus aureus* [402]. A recent study also suggests that the absence of a novel aquaporin (AqpC) reduces pneumococcal autolysis and, consequently, Ply release [403]. Remarkably, the deletion of a*qpC* does not alter the transcription of *lytA.* Thus, Ply secretion is likely regulated by multiple mechanisms that may work together to promote disease progression [115].

In addition to Ply, pneumococci produce several nonclassical cell surface proteins, including the metabolic enzymes triose phosphate isomerase (TpiA) and glyceraldehyde-3-phosphate dehydrogenase (GAPDH). These proteins, which lack both the LPXTG motif and a signal peptide, are nonetheless surface-exposed and secreted by the bacteria [172,404]. The primary role of TpiA is the reversible isomerization of glyceraldehyde-3-phosphate and dihydroxyacetone phosphate. However, it also functions as a moonlighting protein [405,406,407], exhibiting various additional roles [408]. TpiA is released extracellularly through LytA-dependent autolysis, where it binds to host plasminogen and promotes activation of plasmin, a plasma serine protease, potentially aiding bacterial invasion by degrading the extracellular matrix [409,410]. Similarly, GAPDH in *S. pneumoniae* also functions as a plasminogen-binding protein [411]. This protein plays a crucial role in the bacterium’s ability to cross endothelial and epithelial barriers [412] and is released during LytA-dependent autolysis in a subset of the bacterial population [413].

The PepO protease performs various important functions in pneumococcal virulence [414]. Similarly to TpiA, PepO lacks membrane-spanning domains, such as the LPXTG motif region—a hallmark of many surface-exposed proteins—as well as typical signal sequences. Nevertheless, PepO appears to be present on the cell surface and in culture supernatants, indicating it is a secreted protein [415]. While the precise mechanisms underlying the surface localization, secretion, or presence in the culture supernatant of PepO remain unclear, its localized concentration is anticipated to increase several-fold during pneumococcal autolysis. Comparable results have been published with another moonlighting protein, elongation factor Tu [416], which has been reported to bind human complement inhibitors Factor H, FHL-1, CFHR1, and also the proenzyme plasminogen [417,418].

### 6.3. Other Roles of LytA in Pneumococcal Pathogenesis

A new physiological function of LytA named capsular shedding has been described [419]. Rather than inducing autolysis, LytA, distributed circumferentially around the cell, enhances bacterial survival and facilitates rapid capsule shedding in response to cathelicidin LL-37, a cationic antimicrobial peptide (CAMP) present in the human epithelium [420]. Capsule shedding enhances bacterial resistance to this innate defense molecule and permits a close interaction of the bacteria with host cells, leading to the successful initiation of infection. CAMPs are among the relatively few epithelial surface molecules with direct microbicidal activity, with LL-37 being the only cathelicidin identified in humans (for a recent review, see [421]). Previous studies have independently demonstrated that pneumococci can shed their capsules during epithelial cell adherence and invasion [422] and that anionic bacterial capsules act as decoys to evade CAMPs [423]. The newly discovered role of LytA in facilitating capsule removal to counteract CAMPs may help explain why nearly all clinical isolates of pneumococci retain this enzyme, despite the strong selective pressure exerted by antibiotics.

In addition to cathelicidins, defensins represent another major group of mammalian CAMPs. These small, multifunctional cationic peptides play a crucial role in host defense [424,425]. It has been reported that the presence or absence of LytA does not affect encapsulated pneumococci. However, in nonencapsulated strains, *lytA* mutants were more susceptible to antimicrobial peptides than the *lytA*^+^ parental strain [426].

*Streptococcus pneumoniae* DNA released during LytA-dependent autolysis triggers the induction of Krüppel-like transcription factor 4 (KLF4) in human lung epithelial cells through a TLR-9-dependent mechanism [427,428]. As a member of the KLF family, KLF4 plays a crucial regulatory role in both physiological and pathological processes, including pneumonia [429,430]. Upon induction, KLF4 binds to the IL-10 promoter, fostering an anti-inflammatory response. In *S. pneumoniae*-infected polymorphonuclear neutrophils; however, KLF4 increases the expression of pro-inflammatory cytokines while decreasing the release of anti-inflammatory cytokines like IL-10 [431,432,433]. The release of potent pro-inflammatory mediators is vital for mounting a robust defense against infection; however, excessive inflammation can result in severe tissue injury. This balance is particularly critical in severe pneumococcal pneumonia, where the interplay between an effective inflammatory response to eliminate pneumococci and the preservation of organ function determines disease outcomes [434,435]. KLF4 functions as a counter-regulatory transcription factor in pneumococcal-induced pro-inflammatory activation of lung epithelial cells, potentially mitigating lung hyperinflammation and preventing subsequent organ failure.

Macrophages contain acidic phagolysosomes that play a crucial role in digesting and clearing invading bacteria. The low pH within these lysosomes facilitates bacterial degradation and ultimately leads to the death of the engulfed bacteria. Acidic stress can induce autolysis in *S. pneumoniae* when incubated at pH 5.9 [436]. While autolysis triggered by competence development (at pH 7.8) is regulated by the two-component system ComDE, no evidence of increased *lytA* transcription under acidic conditions was found [436], in agreement with a previous study using microarray analysis [437]. Additionally, assays using Doc-induced autolysis have demonstrated that LytA levels remained unchanged during acidic exposure [341]. It has been proposed that autolysis in acidic conditions may be driven by the translocation of LytA from the intracellular to the extracellular compartment after acidic pH stalled bacterial growth [436]. Apparently, under acidic conditions, the surface-expressed LytA is down-regulated by the CiaRH TCS via a CSP-independent ComE pathway [438]. Moreover, the F_0_F_1_-ATPase, a proton pump responsible for maintaining intracellular pH [28], is essential for the survival of *S. pneumoniae* within macrophages [436]. Additional research has also shown that, under acidic conditions, a StkP/ComE-independent pathway regulates the expression of over 100 genes involved in various cellular processes [439]. Mutants lacking StkP exhibit morphological abnormalities, impaired growth, defects in cell division, and enhanced LytA-dependent autolysis. Additionally, these mutants demonstrate reduced tolerance to stress, including acidic environments. In addition, it has been recently suggested that the SirRH (TCS01) is essential for the acidic stress response of *S. pneumoniae* [440].

- LytA is a critical virulence factor in *S. pneumoniae*, with its role validated across diverse animal infection models. It facilitates immune evasion by inhibiting complement activation, recruiting host regulatory proteins, and limiting phagocytosis. LytA also binds to PSGL-1, a host selectin ligand, promoting bacterial clearance during systemic infection. Functionally, LytA mediates the release of intracellular virulence determinants, including pneumolysin (Ply) and non-classically secreted surface-associated proteins which contribute to tissue invasion and host immune modulation. Moreover, LytA is involved in capsule shedding. Autolysis-dependent DNA release activates KLF4, a transcription factor involved in modulating host pro- and anti-inflammatory responses. Under acidic stress, such as within macrophage phagolysosomes, LytA activity supports bacterial survival through pH-dependent regulation—likely mediated by the CiaRH and SirRH two-component systems—and potentially via translocation rather than increased transcription. Altogether, LytA functions as a multifaceted effector of pneumococcal virulence, contributing to immune evasion, toxin release, host interaction, and environmental adaptation.

## 7. Therapeutic Perspectives

With the growing global challenge of antibiotic resistance, phage endolysins are being explored as potential alternatives or adjuncts to traditional antimicrobials [441]. Endolysins can be encoded by both virulent and temperate phages. To date, only a limited number of virulent phages capable of infecting *S. pneumoniae* have been identified. However, prophages (named PPH for Pneumococcal Prophage) are prevalent in pneumococcal genomes. Notably, including both full-length and partial PPH sequences, a substantial proportion (80–90%) of over 4000 putative pneumococcal isolates contain PPHs. The majority of these prophages encode LytA-like lysins of the amidase_2 family [442]. Interestingly, the phage genes coding for these lysins (*lytA*_PPH_) are identical in length and exhibit high sequence similarity (85–92% identity) to *lytA_Spn_*. Surprisingly, only PPH endolysins unrelated to the host LytA NAM-amidase—such as Cpl-1/Cpl-7-like lysozymes or members of the Pal amidase_5 protein family—have been tested in vitro or in vivo so far [443,444]. One possible explanation for this condition is the widely accepted idea that LytA*_Spn_* binds to the cell wall during the exponential growth phase without inducing lysis, while *S. pneumoniae* becomes susceptible to extracellular LytA only in the stationary phase or when cell wall synthesis is inhibited [341,445]. Only three studies have examined the potential therapeutic activity of LytA*_Spn_* in comparison to various phage lysins. In vitro experiments using two serotype 3, PEN-susceptible strains and two PEN-resistant *S. pneumoniae* clinical isolates, were treated with different combinations of LytA, Cpl-1 (a lysozyme encoded by the virulent phage Cp-1), Pal (a phage-encoded lysin of the amidase_5 family), and/or antibiotics (cefotaxime and moxifloxacin). The results demonstrated that LytA_S*pn*_ exhibits greater bactericidal activity than Cpl-1 and Pal [446]. Furthermore, time-kill experiments in a mouse model of peritonitis-sepsis revealed that intraperitoneal therapy with LytA*_Spn_* or high-dose Cpl-1 significantly reduced peritoneal bacterial counts (>5 log₁₀ colony-forming units/mL) compared to the controls. Notably, after intravenous administration, LytA proved to be the most effective treatment [447]. Additionally, *S. pneumoniae* LytA has also been shown to be the most potent enzyme in disrupting in vitro pneumococcal biofilms when compared to the phage lysins Cpl-1, Cpl-7, and Pal [448]. Notably, Ejl (NP_945312.1), a LytA*_Spn_* homolog (86% identity/93% similarity) consisting of 316 aa residues and encoded by the *S. mitis* prophage EJ-1 [449,450], produced a biofilm disintegration of approximately 80%, a level comparable to that caused by the pneumococcal LytA [448].

Another complementary possibility resides in the use of molecules capable of triggering the destructive potential of LytA during IPD. A study demonstrated that various synthetic antimicrobial peptides exhibit significant therapeutic potential in murine models of septicemia and pneumonia [451]. Notably, the combination of one of such peptides, DM3, with PEN enhanced treatment outcomes through therapeutic synergism. Interestingly, in silico molecular docking analyses indicated that DM3 has a strong binding affinity for the LytA autolysin [451]. In addition, cationic ultrashort lipopeptides (USLPs) have recently emerged as promising antimicrobial agents for combating MDR bacteria. Notably, both anionic and neutral zwitterionic USLPs have demonstrated potent, LytA-dependent antimicrobial activity against *S. pneumoniae* [452]. Interestingly, a recent report has shown that extracts from *Lawsonia inermis*, a medicinal plant used in Indonesia, exhibited a potent antipneumococcal activity by producing bacterial lysis, antibiofilm activity, and PG disruption by the increase in the synthesis of *lytA* [453]. Moreover, *Coptis rhizome*, also known as *Huang Lian* in traditional Chinese medicine and commonly used for respiratory infections, is derived from the dried rhizome of *Coptis chinensis* Franch. Ethanolic (70%) extracts exhibited lytic activity against both planktonic and biofilm-grown *S. pneumoniae* cells, regardless of MDR. Notably, treatment with these extracts led to the upregulation of *lytA*, suggesting a potential mechanism involving autolysin activation [454].

Furthermore, 2CCA-1, an alkylated dicyclohexyl carboxylic acid and a polyunsaturated fatty acid mimetic has been identified as a novel inducer of autolysin-mediated lysis in *S. pneumoniae* [455]. It appears to be metabolized similarly to fatty acids and incorporated into phospholipid biosynthesis, leading to the accumulation of toxic phospholipid species and subsequent autolysis. This finding was not entirely unexpected, as the lytic effect of fatty acids on *S. pneumoniae* had been well documented in early studies [456,457].

Miltefosine (hexadecylphosphocholine), the first oral drug approved for the treatment of visceral leishmaniasis, has been found to induce pneumococcal autolysis by promoting the uncontrolled activation of LytA [458]. A similar effect was later observed in *Bacillus subtilis* [459]. Interestingly, miltefosine does not appear to affect membrane ordering or packing but instead alters the transport of small molecules across the membrane [460].

Ceragenins are a novel class of agents designed to mimic the function of endogenous antimicrobial peptides, making them promising candidates for the development of new antibacterial compounds [461]. In particular, ceragenin CSA-13, a cationic steroid, exhibits concentration-dependent bactericidal and bacteriolytic activity against pathogenic streptococci, including MDR *S. pneumoniae*. The lytic effect of CSA-13 is attributed to its activation of the major autolysin LytA [462]. Subsequently, CSA-13A demonstrated a bactericidal activity against various bacteria stronger than that produced by cathelicidin LL-37. Apparently, bacterial exposure to CSA-13 did not result in the emergence of resistance [463]. More recently, CSA-13 has been reported as a highly effective antimicrobial agent with activity against a broad range of bacterial species, including MDR Gram-negative rods [464,465].

- LytA shows strong therapeutic potential as an antimicrobial agent. Compared to phage lysins, LytA demonstrates superior bactericidal and antibiofilm activity in *S. pneumoniae*. Various compounds—including antimicrobial peptides, plant extracts, lipid mimetics, and ceragenins—exert LytA-dependent lytic effects, either by activating the enzyme or inducing its expression. These findings highlight LytA as a promising target for novel treatments against MDR pneumococcal isolates.

## 8. Evolutionary Considerations

Notably, *lytA*_PPH_ genes exhibit a strong similarity to *lytA_Spn_* [442]. Earlier studies reported that while most *lytA_Spn_* genes could be categorized into two primary families (Fam_A and Fam_B), *lytA*_PPH_ alleles displayed significantly greater diversity [466]. Sequence comparisons between *lytA_Spn_* and *lytA*_PPH_ alleles suggested that recombination events occurred between host DNA and prophages within the *lytA* gene [466,467]. Indeed, laboratory experiments documented evidence of recombination involving three distinct *lytA_Spn_* alleles and *hbl*, the *lytA*_PPH_ gene of prophage HB-3 [468]. Furthermore, recent sequence analyses of various PPH families have revealed that PPH090 represents a group of prophages capable of integrating into the *lytA_Spn_* gene via recombination, leading to the formation of potentially chimeric (hybrid) *lytA* genes containing sequences of both phage and bacterial origin [442]. Additionally, recombination between prophage and host *lytA* genes has been identified as a significant driver of chromosomal rearrangements in *S. pneumoniae* though the consequences of such genome rearrangements on both bacterial and phage physiology warrants further investigation. Nevertheless, the recombination-to-mutation ratio is >1 in *S. pneumoniae*, indicating that recombination has played a more significant role in the diversification of this species than mutation [469].

- The *lytA* genes encoded by pneumococcal prophages (lytA_PPH_) show high similarity to the host *lytA_Spn_* but exhibit greater sequence diversity. Studies have shown that recombination events between prophage and host DNA within the *lytA* locus can produce chimeric genes, contributing to chromosomal rearrangements and genetic diversity in *S. pneumoniae*. This highlights recombination, more than mutation, as a key driver of pneumococcal evolution.

## 9. Future Perspectives

LytA has demonstrated significant potential as a therapeutic agent due to its bactericidal and biofilm-disrupting properties. Its ability to hydrolyze pneumococcal cell walls and efficiently lyse bacterial cells makes it an attractive candidate for antimicrobial therapies, particularly against antibiotic-resistant strains. In this sense, LytA represents a promising alternative (or adjunct) to traditional antibiotics. However, further research is needed to investigate its safety in human models and explore potential synergies with existing antimicrobial therapies. With its strong bactericidal effects, biofilm-targeting capabilities, and potential for genetic optimization, LytA could play a key role in the development of novel anti-pneumococcal therapies.

As already mentioned (Section 1), the extensive diversity of pneumococcal serotypes, along with limited serotype coverage and the replacement of vaccine-covered strains by non-PCV13 variants, continue to present significant challenges. The novel 20-valent PCV, when used alone, is likely to be cost-effective or superior to other adult pneumococcal vaccination strategies, although further studies are needed to confirm its efficacy and impact [470]. In this context, alternatives to capsular polysaccharide-based vaccines are increasingly being explored (for recent reviews, see [471,472,473,474]). These alternatives include not only protein-based vaccines but also whole-cell pneumococcal ones, which may offer potential broad-spectrum protection against IPD. Among the various *S. pneumoniae* proteins that have been evaluated as immunogens—such as Ply, PspA, or PspC—LytA has also been tested, albeit in a limited number of studies and some promising results have been reported in animal models [475,476,477]. In the single instance where LytA was used as an immunogen in humans, a strong immunological response was observed [478]. However, as expected, variations were noted among different age groups. More studies must be performed to test the LytA autolysin for safety, tolerability, and immunogenicity of the future serotype-independent pneumococcal vaccines. In addition, two concerns have been raised regarding LytA as a vaccine candidate [471]. First, *S. pseudopneumoniae* (and some other SMG) possesses a LytA protein similar to that of pneumococcus (see above), potentially making it a target for LytA antibodies and disrupting the microbial balance. Second, secretory IgAs [479] may influence colonization dynamics and could create a favorable niche for other pathogens.

Animal experimentation has played a crucial role in developing treatments and vaccines that have saved countless lives. The use of animal models in pneumococcal vaccine research has both benefits and drawbacks [480,481]. On the positive side, they offer physiological similarity, allow for safety and efficacy assessments, and contribute to the development of effective treatments. However, challenges include inter-species differences, ethical concerns, and limitations in accurately replicating human diseases. Therefore, while animal models remain valuable tools in pneumococcal vaccine development, it is essential to acknowledge their limitations and explore complementary approaches to ensure vaccine safety and effectiveness in healthy humans since *S. pneumoniae* is primarily a human pathogen. Fortunately, human models of pneumococcal nasopharyngeal carriage are currently being tested, with the first experiments being conducted over two decades ago [482]. The Experimental Human Pneumococcal Challenge model enables the evaluation of vaccines by assessing their impact on experimental *S. pneumoniae* colonization [483]. In this model, human volunteers are intranasally inoculated with pneumococci, leading to a stable colonization episode lasting approximately 1–3 weeks at a density similar to natural colonization. Several of these studies are currently ongoing (see ref. [484,485] and references therein). Notably, a number of clinical trials are also testing protein-based vaccines [474,486], yielding promising results.

- LytA is a promising antimicrobial and vaccine candidate due to its strong bactericidal and biofilm-disrupting activity, especially against antibiotic-resistant *S. pneumoniae*. Though effective in animal models and immunogenic in humans, further studies are needed to confirm its safety and efficacy. As polysaccharide vaccines face challenges like serotype replacement, protein-based alternatives—including LytA—are under active investigation. Human challenge models and clinical trials are aiding progress toward serotype-independent pneumococcal vaccines.

## Figures and Tables

**Table 1:** The LytA autolysin and antibiotic tolerance factors.

Factor	Description ^a^	References
LytA deficiency	Failure to express enzymatically active LytA autolysin leads to AT. Only one clinical isolate has been identified as a true *lytA* mutant.	[51,57]
*psaBCAD* mutation	Mutation in *psaBCAD* locus, coding for an ATP-binding cassette Mn^2+^-permease complex, results in failure to synthesize LytA and AT.	[298]
*zmpB* mutation	Mutation in *zmpB*, encoding a Zn^2+^ metalloprotease, results in failure to synthesize LytA and AT.	[299]
Controversial results	Neither *psaBCAD* nor *zmpB* are involved in AT.	[300,301,302]
*clpC* mutation	Mutation in *clpC* leads to long chains of cells and failure to lyse after PEN or VAN reatment. Effects are strain-dependent.	[303,304]
	*clpC* mutants exhibit a non-tolerant phenotype and do not form long chains of cells.	[305,306]
*vncS*/*vncR* mutations	Mutation in *vncS*, encoding a histidine kinase, confers VAN tolerance and extends to other antibiotics like β-lactams, aminoglycosides, and quinolones. No detectable changes in LytA production. Mutation in *vncR* does not lead to a tolerant phenotype.	[131]
Pep^27^ secretion ^b^	Pep^27^ secretion was proposed to trigger multiple cell death mechanisms, but later studies failed to replicate these findings.	[307]
	Other studies did not confirm the contribution of *vncS*, *vex3*, *vncR*, or *pep27* in AT.	[308,309]
Other factors	TCS03, TCS11, and CiaRH and carbohydrate metabolism-related proteins may also be involved in VAN tolerance	[310,311]
PtvR regulation	PtvR regulates the *ptvRABC* operon, enhancing VAN tolerance. PtvR mutants exhibited reduced susceptibility to VAN. No effect on PEN tolerance.	[312]
Cid (Tol) phenotype	Cid⁻ (Tol^−^) mutants showed reduced lysis in response to cell wall-active antibiotics, suggesting two killing mechanisms: one LytA-dependent and one LytA-independent.	[74,313,314]
*murMN* operon	Mutants lacking *murMN* displayed increased susceptibility to lysis when exposed to antibiotics, affecting PG structure.	[315,316,317]
*O*-acetylation of PG	*O*-acetylation of PG may modify stationary-phase lysis.	[318,319]

^a^ AT, antibiotic tolerance. ^b^ Pep^27^ is a 27-aa secreted peptide of *S. pneumoniae* that appears to function as a major virulence factor.

**Table 2:** Virulence of *lytA* mutants vs. *lytA*^+^ strains of *S. pneumoniae* tested in animal models of infection.

Host	Disease	Route ^a^	Strain Tested (Serotype)	Virulence ^b^	References
Mouse	Bacteremia	ip	D39 (2); ND (3)	↓	[371,372]
	Pneumonia	in	D39 (2)	↓	[371,373,374]
	Bacteremia	ip; iv	WU2 (3)	No change	[375]
	Bacteremia	ip	A66 (3); A112 (6A)	No change	[376]
	Bacteremia	ip	D39 (2); GB05 (3)	↓	[377]
	Bacteremia/pneumonia	in; ip	D39 (2); 1515/97 (6B); S3 (23F);	↓	[378,379]
	Bacteremia/pneumonia	in; it; iv	D39 Xen7 (2)	↓	[380]
	Meningitis	ic	D39 (2)	↓	[381]
Adult zebrafish	Bacteremia/meningitis	im; ip	TIGR4 (4)	↓	[382]
Chinchilla	Otitis media	me	WT3 (3)	↓	[92]
Rat	Nasal colonization	in	D39 (2); WT (3)	No change	[383]
	Endophthalmitis	ivt	D39 (2)	↓	[384]

^a^ ic, intracisternal; im, intramuscular; in, intranasal; ip, intraperitoneal; it, intratracheal; iv, intravenous; ivt, intravitreal; me, middle ear. ^b^ ↓, indicates reduced virulence.

## Data Availability

All data generated or analyzed during this study are included in this published article.

## Supplementary Materials

The following supporting information can be downloaded at: https://www.mdpi.com/article/10.3390/microorganisms13040827/s1, Table S1: Accession numbers of genes discussed in this review across four *S. pneumoniae* strains.

## Abbreviations

The following abbreviations are used in this manuscript:

ABC	ATP-binding cassette
CAMP	Cationic antimicrobial peptide
CBD	Choline-binding domain
CBP	Choline-binding protein
CBR	Choline-binding repeat
CBS	Choline-binding site
CDM	Chemically defined medium
CSF	Cerebrospinal fluid
CSP	Competence-stimulating peptide
csRNA	CiaR-dependent sRNA
Doc	Sodium deoxycholate
DPC	Dodecylphosphocholine
EAD	Enzymatically active domain
eDNA	Extracellular DNA
IPD	Invasive pneumococcal disease(s)
LTA	Lipoteichoic acid
MDR	Multidrug-resistant
MIC	Minimum inhibitory concentration
NAM-amidase	*N*-acetylmuramoyl-l-alanine amidase
ORF	Open reading frame
P-Cho	Phosphocholine
PCV	Pneumococcal conjugate vaccine(s)
PEN	Penicillin
PG	Peptidoglycan
PGRP	PG recognition protein
PPH	Pneumococcal prophage
SMG	Streptococci of the Mitis group
sORF	Small ORF
sRNA	Small RNA
TA	Teichoic acid
TCS	Two-component regulatory system
TSS	Transcription start site
USLP	Ultrashort lipopeptides
VAN	Vancomycin
WHO	World Health Organization
WTA	Wall teichoic acid
