# Supplementary material for: Structure, Function, and Regulation of LytA: The N-Acetylmuramoyl-l-alanine Amidase Driving the “Suicidal Tendencies” of Streptococcus pneumoniae—A Review
Source: Microorganisms. 2025 Apr 5;13(4):827. doi: 10.3390/microorganisms13040827 (PMC12029793; doi:10.3390/microorganisms13040827)
Supplement: Supplementary file 1 [file microorganisms-13-00827-s001.zip › microorganisms-3549316-supplementary.pdf]

Supplementary Table S1. Accession numbers of genes discussed in this review across four *S. pneumoniae* strains.

Gene (orientation) [Protein]	TIGR4 (2,160,842 bp)		R6 (2,038,615 bp)		D39 (2,046,116 bp)		D39V (2,046,572 bp)	
	NC_003028.3	AE005672.3	NC_003098.1	AE007317.1	NC_008533.2	CP000410.2	NZ_CP027540.1	CP027540.1
ftsH	SP_RS00065	SP_0013	SPR_RS00060	spr0012	SPD_RS00060	SPD_0013	SPV_RS00060	SPV_0013
comX1	SP_RS00070	SP_0014	SPR_RS00065	spr0013	SPD_RS00065	SPD_0014	SPV_RS00065	SPV_0014
comW	SP_RS00130	SP_0018	SPR_RS00110	spr0020	SPD_RS00110	SPD_0023	SPV_RS00110	SPV_0023
ptvC (/c)	SP_RS00490	SP_0097	SPR_RS00475	spr0086	SPD_RS00500	SPD_0093	SPV_RS00500	SPV_0093
ptvB (/c)	SP_RS00495	SP_0098	SPR_RS00480	spr0087	SPD_RS00505	SPD_0094	SPV_RS00505	SPV_0094
ptvA (/c)	SP_RS00500	SP_0099	SPR_RS00485	spr0088	SPD_RS00510	SPD_0095	SPV_RS00510	SPV_0095
ptvR (/c)	SP_RS00505	SP_0100	SPR_RS00490	spr0089	SPD_RS00515	SPD_0096	SPV_RS00515	SPV_0096
pspA	SP_RS00595	SP_0117	SPR_RS00665	spr0121	SPD_RS00695	SPD_0126	SPV_RS00695	SPV_0126
cibC (/c)	SP_RS00630	SP_0122		spr0126		SPD_0131		SPV_2109
cibB (/c)	SP_RS00635	SP_0124	SPR_RS00695	spr0127	SPD_RS00735	SPD_0132	SPV_RS00735	SPV_0132
cibA (/c)	SP_RS00640	SP_0125	SPR_RS00700	spr0128	SPD_RS00740	SPD_0133	SPV_RS00740	SPV_0133
spxA2	SP_RS00930	SP_0189	SPR_RS00930	spr0173	SPD_RS00990	SPD_0178	SPV_RS00990	SPD_0178
pbp2X	SP_RS01640	SP_0336	SPR_RS01590	spr0304	SPD_RS01675	SPD_0306	SPV_RS01675	SPV_0306
luxS (/c)	SP_RS01655	SP_0340	SPR_RS01605	spr0308	SPD_RS01690	SPD_0309	SPV_RS01690	SPV_0309
cps4A/*cps2A*	SP_RS01690	SPD_0346	SPR_RS01630	spr0314	SPD_RS01715	SPD_0315	SPV_RS01715	SPV_0315
liaF (*vraT*)	SP_RS01890	SP_0385	SPR_RS01775	spr0342	SPD_RS01900	SPD_0350	SPV_RS01900	SPV_0350
liaS (*vraS*)	SP_RS01895	SP_0386	SPR_RS01780	spr0343	SPD_RS01905	SPD_0351	SPV_RS01905	SPV_0351
liaR (*vraR*)	SP_RS01900	SP_0387	SPR_RS01785	spr0344	SPD_RS01910	SPD_0352	SPV_RS01910	SPV_0352
cbpF (*pcpC; cbpC*)	SP_RS01930	SP_0391	SPR_RS01815	spr0351	SPD_RS01940	SPD_0357	SPV_RS01940	SPV_0357
rrgB	SP_RS02285	SP_0463						
pnp	SP_RS02910	SP_0588	SPR_RS02615	spr0516	SPD_RS02755	SPD_0512	SPV_RS02750	SPV_0512
–/vex1	SP_RS02950	SP_0599	SPR_RS02660	spr0524	SPD_RS02795	SPD_0521	SPV_RS02790	SPV_0521
vex2	SP_RS02955	SP_0600	SPR_RS02665	spr0525	SPD_RS02800	SPD_0522	SPV_RS02795	SPV_0522
vex3	SP_RS02960	SP_0601	SPR_RS02670	spr0526	SPD_RS02805	SPD_0523	SPV_RS02800	SPV_0523
pep27	SP_RS13110	SP_0602	SPR_RS10635	spr0527	SPD_RS11415	–	SPV_RS11440	SPV_2576
vncR	SP_RS02965	SP_0603	SPR_RS02675	spr0528	SPD_RS02810	SPD_0524	SPV_RS02805	SPV_0524
vncS	SP_RS02970	SP_0604	SPR_RS02680	spr0529	SPD_RS02815	SPD_0525	SPV_RS02810	SPV_0525
murM	SP_RS03020	SP_0615	SPR_RS02730	spr0540	SPD_RS02870	SPD_0535	SPV_RS02865	SPV_0535
murN	SP_RS03025	SP_0616	SPR_RS02735	spr0541	SPD_RS02875	SPD_0536	SPV_RS02870	SPV_0536
rr09	SP_RS03245	SP_0661	SPR_RS02930	spr0578	SPD_RS03105	SPD_0574	SPV_RS03100	SPV_0574

hk09	SP_RS03250	SP_0662	SPR_RS02935	spr0579	SPD_RS03110	SPD_0575	SPV_RS03105	SPV_0575
zmpB	SP_RS03260	SP_0664	SPR_RS02945	spr0581	SPD_RS03120	SPD_0577	SPV_RS03115	SPV_0577
lctO (/c)	SP_RS03500	SP_0715	SPR_RS03160	spr0627	SPD_RS03345	SPD_0621	SPV_RS03340	SPV_0621
spxB	SP_RS03575	SP_0730	SPR_RS03235	spr0642	SPD_RS03430	SPD_0636	SPV_RS03425	SPV_0636
ciaR	SP_RS03905	SP_0798	SPR_RS03560	spr0707	SPD_RS03775	SPD_0701	SPV_RS03770	SPV_0701
ciaH	SP_RS03910	SP_0799	SPR_RS03565	spr0708	SPD_RS03780	SPD_0702	SPV_RS03775	SPV_0702
spr0810	SP_RS04495	SP_0910	SPR_RS04070	spr0810	SPD_RS04305	SPD_0803	SPV_RS04295	SPV_0803
pavA (/c)	SP_RS04790	SP_0966	SPR_RS04355	spr0868	SPD_RS04595	SPD_0854	SPV_RS04585	SPV_0854
whyD	SP_RS04930	SP_0994	SPR_RS04495	spr0890	SPD_RS04740	SPD_0880	SPV_RS04730	SPV_0880
rpoD (*sigA*) (/c)	SP_RS05305	SP_1073	SPR_RS04905	spr0979	SPD_RS05160	SPD_0958	SPV_RS05180	SPV_0958
psr (/c)	SP_RS06705	SP_1368	SPR_RS06130	spr1226	SPD_RS06425	SPD_1202	SPV_RS06420	SPV_1202
tenA (/c)	SP_RS06880	SP_1404	SPR_RS06300	spr1261	SPD_RS06600	SPD_1235	SPV_RS06595	SPV_1235
spxA1 (/c)	SP_RS06885	SP_1405	SPR_RS06305	spr1262	SPD_RS06605	SPD_1236	SPV_RS06600	SPV_1236
tuf (/c)	SP_RS07325	SP_1489	SPR_RS06690	spr1343	SPD_RS07015	SPD_1318	SPV_RS07010	SPV_1318
aqpC (*gla*) (/c)	SP_RS07340	SP_1491	SPR_RS06695	spr1344	SPD_RS07025	SPD_1320	SPV_RS07020	SPV_1320
– (/c) [Atpε]	SP_RS07420	SP_1507	SPR_RS06765	spr1359	SPD_RS07105	SPD_1334	SPV_RS07100	SPV_1334
*atp*D *(/c)* [Atpβ]	SP_RS07425	SP_1508	SPR_RS06770	spr1360	SPD_RS07110	SPD_1335	SPV_RS07105	SPV_1335
– (/c) [Atpγ]	SP_RS07430	SP_1509	SPR_RS06775	spr1361	SPD_RS07115	SPD_1336	SPV_RS07110	SPV_1336
atpA (/c) [Atpα]	SP_RS07435	SP_1510	SPR_RS06780	spr1362	SPD_RS07120	SPD_1337	SPV_RS07115	SPV_1337
– (/c) [Atpδ]	SP_RS07440	SP_1511	SPR_RS06785	spr1363	SPD_RS07125	SPD_1338	SPV_RS07120	SPV_1338
atpF (/c) [AtpB]	SP_RS07445	SP_1512	SPR_RS06790	spr1364	SPD_RS07130	SPD_1339	SPV_RS07125	SPV_1339
atpB (/c) [AtpA]	SP_RS07450	SP_1513	SPR_RS06795	spr1365	SPD_RS07135	SPD_1340	SPV_RS07130	SPV_1340
– (/c) [AtpC]	SP_RS07455	SP_1514	SPR_RS06800	spr1366	SPD_RS07140	SPD_1341	SPV_RS07135	SPV_1341
lytC (/c)	SP_RS07755	SP_1573	SPR_RS07105	spr1431	SPD_RS07460	SPD_1403	SPV_RS07455	SPV_1403
tpiA (/c)	SP_RS07760	SP_1574	SPR_RS07110	spr1432	SPD_RS07465	SPD_1404	SPV_RS07460	SPV_1404
codY (/c)	SP_RS07810	SP_1584	SPR_RS07155	spr1439	SPD_RS07505	SPD_1412	SPV_RS07500	SPV_1412
sirH (/c)	SP_RS08045	SP_1632	SPR_RS07325	spr1473	SPD_RS07685	SPD_1445	SPV_RS07680	SPV_1445
sirR (/c)	SP_RS08050	SP_1633	SPR_RS07330	spr1474	SPD_RS07690	SPD_1446	SPV_RS07685	SPV_1446
lipA	SP_RS08055	SP_1634	SPR_RS07335	spr1475	SPD_RS07695	SPD_1447	SPV_RS07690	SPV_1447
pepO (/c)	SP_RS08130	SP_1647	SPR_RS07405	spr1491	SPD_RS07765	SPD_1460	SPV_RS07760	SPV_1460
psaB	SP_RS08135	SP_1468	SPR_RS07410	spr1492	SPD_RS07770	SPD_1461	SPV_RS07765	SPV_1461
psaC	SP_RS08140	SP_1469	SPR_RS07415	spr1493	SPD_RS07775	SPD_1462	SPV_RS07770	SPV_1462
psaA	SP_RS08145	SP_1650	SPR_RS07420	spr1494	SPD_RS07780	SPD_1463	SPV_RS07775	SPV_1463
psaD (*tpx*)	SP_RS08150	SP_1651	SPR_RS07425	spr1495	SPD_RS07785	SPD_1464	SPV_RS07780	SPV_1464

ftsZ (/c)	SP_RS08230	SP_1666	SPR_RS07490	spr1510	SPD_RS07860	SPD_1479	SPV_RS07855	SPV_1479
nanB (/c)	SP_RS08335	SP_1687	SPR_RS07590	spr1531	SPD_RS07965	SPD_1499	SPV_RS07960	SPV_1499
nanA (/c)	SP_RS08365	SP_1693	SPR_RS07615	spr1536	SPD_RS07990	SPD_1504	SPV_RS07985	SPV_1504
stkP (/c)	SP_RS08570	SP_1732	SPR_RS07820	spr1577	SPD_RS08205	SPD_1542	SPV_RS08200	SPV_1542
phpP (/c)	SP_RS08575	SP_1733	SPR_RS07825	spr1578	SPD_RS08210	SPD_1543	SPV_RS08205	SPV_1543
mgrA (/c)	SP_RS08935	sp_1800	SPR_RS08055	spr1622	SPD_RS08460	SPD_1587	SPV_RS08455	SPV_1587
tacL (/c)	SP_RS09400	SP_1893	SPR_RS08490	spr1708	SPD_RS08920	SPD_1672	SPV_RS08915	SPV_1672
(/c)	SP_RS09665	SP_1922	SPR_RS08745	spr1738	SPD_RS09175	SPD_1725	SPV_RS09170	SPV_1725
rio87 (/c)								SPV_2545
ply (/c)	SP_RS09670	SP_1923	SPR_RS08750	spr1739	SPD_RS09185	SPD_1726	SPV_RS09180	SPV_1726
(/c)	SP_RS09675	SP_1924	SPR_RS08755	spr1740	SPD_RS09190	SPD_1727	SPV_RS09185	SPV_1727
(/c)	SP_RS09680	SP_1925	SPR_RS08760	spr1741	SPD_RS09195	SPD_1728	SPV_RS09190	SPV_1728
(/c)	SP_RS09685	SP_1926	SPR_RS08765	spr1742	SPD_RS09200	SPD_1729	SPV_RS09195	SPV_1729
lytA (/c)	SP_RS09740	SP_1937	SPR_RS08815	spr1754	SPD_RS09250	SPD_1737	SPV_RS09245	SPV_1737
rio88 (/c)								SPV_2546
dinF (/c)	SP_RS09745	SP_1939	SPR_RS08820	spr1756	SPD_RS09255	SPD_1738	SPV_RS09250	SPB_1738
recA (/c)	SP_RS09750	SP_1940	SPR_RS08825	spr1757	SPD_RS09265	SPD_1739	SPV_RS09260	SPV_1739
cinA (/c)	SP_RS09755	SP_1941	SPR_RS08830	spr1758	SPD_RS09270	SPD_1740	SPV_RS09265	SPV_1740
lytR (/c)	SP_RS09760	SP_1942	SPR_RS08835	spr1759	SPD_RS09275	SPD_1741	SPV_RS09270	SPV_1741
comM (/c)	SP_RS09775	SP_1945	SPR_RS08850	spr1762	SPD_RS09290	SPD_1744	SPV_RS09285	SPV_1744
comX2 (/c)	SP_RS10155	SP_2006	SPR_RS09210	spr1819	SPD_RS09675	SPD_1818	SPV_RS09670	SPV_1818
gap (/c) (GAPDH)	SP_RS10185	SP_2012	SPR_RS09240	spr1825	SPD_RS09705	SPD_1823	SPV_RS09700	SPV_1823
adr (/c)	SP_RS10400	SP_2057	SPR_RS09450	spr1868	SPD_RS09915	SPD_1867	SPV_RS09915	SPV_1867
dltA (/c)	SP_RS11100	SP_2176	SPR_RS10105	spr1982	SPD_RS10600	SPD_2005	SPV_RS10600	SPV_2005
clpC (/c)	SP_RS11210	SP_2194	SPR_RS10185	spr1999/spr2000	SPD_RS10700	SPD_2022	SPV_RS10700	SPV_2022
cbpD (/c)	SP_RS11240	SP_2201	SPR_RS10215	spr2006	SPD_RS10730	SPD_2028	SPV_RS10730	SPV_2028
comE (/c)	SP_RS11425	SP_2235	SPR_RS10400	spr2041	SPD_RS10920	SPD_2063	SPV_RS10915	SPV_2063
comD (/c)	SP_RS11430	SP_2236	SPR_RS10405	spr2042	SPD_RS10925	SPD_2064	SPV_RS10920	SPV_2064
comc (/c)	SP_RS11435	SP_2237	SPR_RS10410	spr2043	SPD_RS10930	SPD_2065	SPV_RS10925	SPV_2065
htrA	SP_RS11450	SP_2239	SPR_RS10425	spr2045	SPD_RS10945	SPD_2068	SPV_RS10940	SPV_2068

[a] The *lytA* gene is highlighted in green. In red, pseudogenes. /c means that the sequence corresponds to the complementary strand.
